# Structural basis for the procoagulant activity of *Staphylococcus aureus* staphylocoagulase

**DOI:** 10.1515/hsz-2025-0253

**Published:** 2026-08-20

**Authors:** Pablo Fuentes-Prior, Ashoka A. Maddur, Peter Panizzi, David Gailani, Ingrid M. Verhamme

**Affiliations:** Department of Biochemistry and Molecular Biomedicine, Faculty of Biology, Institute of Biomedicine of the University of Barcelona (IBUB), University of Barcelona (UB), E-08028, Barcelona, Spain; FUJIFILM Biotechnologies, College Station, TX, 77845, USA; Department of Drug Discovery and Development, Harrison College of Pharmacy, Auburn University, Auburn, AL, 36849-5506, USA.; Department of Pathology, Microbiology and Immunology, Vanderbilt University Medical Center, Nashville, TN, 37232, USA; Department of Pathology, Microbiology and Immunology, Vanderbilt University Medical Center, Nashville, TN, 37232, USA

**Keywords:** cofactor-assisted activation, coagulation, fibrinogen, molecular modeling, *Staphylococcus aureus*, trypsin-like serine proteinases

## Abstract

Staphylocoagulase (SC), a crucial virulence protein secreted by several strains of the major pathogen *Staphylococcus aureus*, functions as a potent inducer of blood clotting. SC binds its zymogen target, prothrombin (ProT) with subnanomolar affinity, allosterically inducing a thrombin-like active site in the bound zymogen. The resulting SC·(Pro)T* active complexes effectively convert fibrinogen (Fbg) into clot-forming fibrin (Fbn). We previously reported crystal structures of the N-terminal SC domains, responsible for ProT binding and activation, and characterized the mechanism of cofactor-induced zymogen activation in detail. However, the lack of three-dimensional (3D) structures for the C-terminal SC region has hampered a complete understanding of its role in Fbg recognition and cleavage. Here, we present and discuss 3D models of the Fbg-binding SC region. Our results suggest that the major C-terminal domain of SC folds into a single-layer β-sheet structurally similar to the membrane occupation and recognition nexus (MORN) family of tandem repeats. We further propose a folding pathway for this C-terminal SC region that is contingent upon the presence of its substrate, Fbg. These structural and mechanistic insights are critical for understanding SC-mediated fibrin generation, which is highly relevant to heart valve vegetations and biofilm formation during *S. aureus* infection.

## 1 Introduction

*Staphylococcus aureus* is a ubiquitous human commensal and a formidable pathogen, colonizing the nasal mucosa of 20–40 % of the population (Sakr et al. 2018). It is a leading cause of both nosocomial and community-acquired infections, ranging from minor skin abscesses to life-threatening conditions, including pneumonia, endocarditis, and sepsis (Lowy 1998; Panizzi et al. 2004). The rise of methicillin-resistant *S. aureus* (MRSA) represents a global health crisis; it is now the second leading cause of death associated with antimicrobial resistance (Antimicrobial Resistance Collaborators 2022), with hospitalizations and mortality rates continuing to escalate (Biggs et al. 2025; Dean 2025). The clinical burden is substantial: in the U.S. alone, staphylococcal infections affect approximately 500,000 hospital patients and contribute to up to 50,000 deaths annually (Goetghebeur et al. 2007; Gordon and Lowy 2008; Schlecht et al. 2015). This crisis underscores the urgent need for a structure-based understanding of staphylococcal infection mechanisms. Furthermore, *S. aureus* is a significant pathogen across a wide range of both domestic and wild animal species, contributing to a major economic impact (Haag et al. 2019; Park and Ronholm 2021).

**Figure 1:**
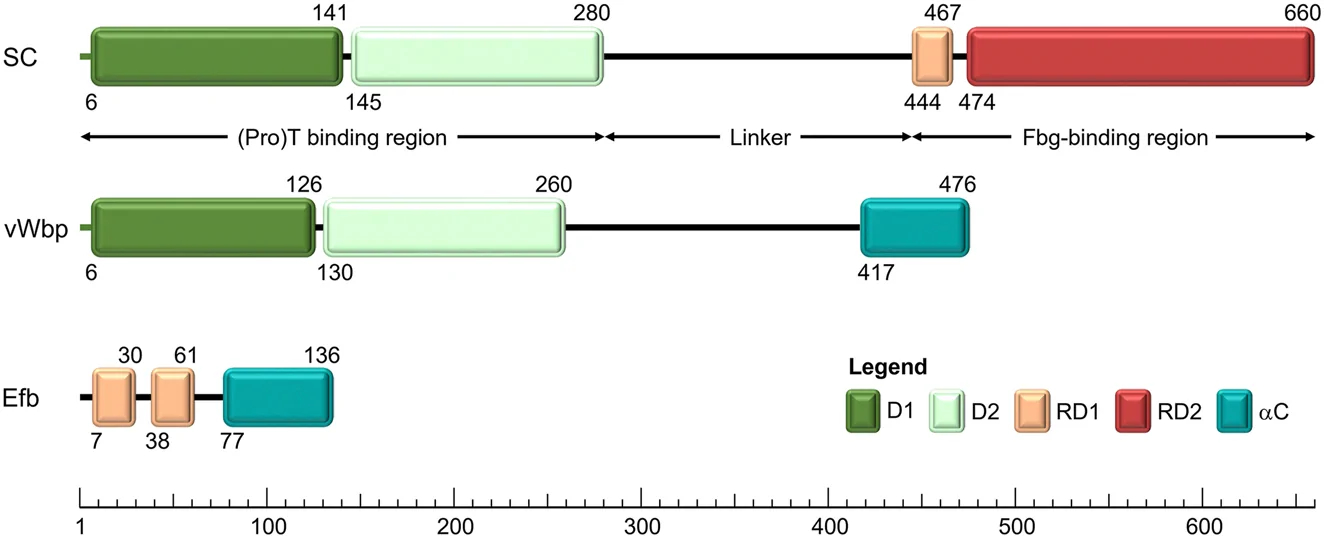
Schematic representation of the domain organization in staphylocoagulase (SC) and related staphylococcal virulence proteins. Domain architecture is shown for the mature secreted proteins from *Staphylococcus aureus* strain Tager 104: SC (GenBank entry AMO52898.1), vWbp (AMO52319.1) and Efb/Fib (AMO51973.1) (Davis et al. 2016). The N-terminal activator peptides of SC and vWbp mimic the canonical Ile^16^ N-terminus of the serine protease domain of the cognate zymogen, prothrombin (ProT). Upon insertion into the ProT activation pocket, these N-terminal peptides – assisted by the α-helical D1 and D2 domains – allosterically stabilize a catalytically active ProT conformation. The C-terminal half of SC comprises two distinct Fbg-binding modules, RD1 and RD2, connected to the N-terminal ProT-binding unit by a long, disordered linker. Notably, the N-terminal region of Efb contains two modules with high structural homology to SC-RD1. The C-terminal αC domain of vWbp interacts specifically with the A1 domain of von Willebrand factor (Bjerketorp et al. 2002), and harbors additional Fbg-binding activity (Thomas et al. 2021). Importantly, this C-terminal domain has been shown to increase vWbp’s affinity for ProT via an allosteric mechanism (Kroh et al. 2009). The structurally related domain in Efb has been shown to bind complement factor C3/C3b instead (Lee et al. 2004a; Lee et al. 2004b).

*Staphylococcus aureus* employs an extensive arsenal of secreted and membrane-associated virulence factors that hijack host physiological systems, most notably the blood coagulation and fibrinolytic cascades (Howden et al. 2023; Liesenborghs et al. 2018; Risser et al. 2022). A central player in this process is the secreted protein, staphylocoagulase (SC). SC-deficient mutants exhibit significantly reduced infectivity, establishing SC as a critical virulence factor (Cheng et al. 2010). Even strains that do not express coagulases benefit from the secreted factor to evade antimicrobials and immune factors of the host (Trivedi et al. 2018). Across several hundred variants, SC shares a conserved domain organization: an N-terminal activator peptide followed by two helical bundles (D1 and D2), a central disordered region, and a variable number of C-terminal repeats with over 90 % identity, preceded by a conserved “pseudo-repeat” (Watanabe et al. 2009) (Figure 1).

The N-terminal regions of SC and its homolog, von Willebrand factor-binding protein (vWbp), bind tightly to α-thrombin and its zymogen, prothrombin (ProT). This interaction induces a proteolytically competent active site in ProT that is structurally and functionally indistinguishable from that of the free proteinase. While the active site of the complexed (Pro)T efficiently cleaves small-molecule substrates, occupancy of the surrounding loops by the bulky D1/D2 domains effectively occludes platforms critical for recognizing macromolecular thrombin interactors, most notably exosite I (Fuentes-Prior et al. 2000; Panizzi et al. 2004; Kroh et al. 2009; Maddur et al. 2020). As a result, (Pro)T complexed with SC is exclusively directed toward generating fibrin (Fbn) from circulating fibrinogen (Fbg). SC and vWbp cooperate to form extensive Fbn biofilms, or “vegetations”, which shield *S. aureus* from immune detection and contribute to antibiotic resistance (Evans et al. 2024; Guggenberger et al. 2012).

We have previously reported the three-dimensional (3D) crystal structures of SC N-terminal domains bound to α-thrombin and the zymogen, prethrombin-2, along with a thorough characterization of the mechanism of cofactor-induced zymogen activation (Friedrich et al. 2003; Kroh et al. 2009; Maddur et al. 2020; Panizzi et al. 2004). However, the structure of SC’s C-terminal half, which contains the major Fbg-binding site, remains unknown. This structural gap complicates the analysis of Fbg recognition and processing by SC·(Pro)T complexes, hindering the rational search for alternative treatments. Here, we address this limitation by presenting highly reliable 3D models of the C-terminal repeat domains of SC and discuss their implications for understanding the mechanism of fibrinogen recognition and processing.

## 2 A structural and functional analysis of SC’s role in fibrinogen processing

### 2.1 The mechanism of proteolysis-independent prothrombin activation

Wolfram Bode and Robert Huber’s pioneer work on the molecular basis of the activation of trypsin-like serine proteinases (TLSPs) highlighted the critical role played by the insertion of the newly generated N-terminal peptide (with the canonical sequence Ile^16^-Val-Gly-Gly), into the main body of the protease (Bode and Huber 1976; Bode et al. 1978; reviewed by Perona and Craik 1995). This N-terminal insertion induces conformational rearrangements that establish the enzyme’s active site (His^57^, Asp^102^ and Ser^195^) upon formation of a critical salt bridge with the carboxylate of Asp194. (The sequences of TLSPs are numbered according to the structure-based chymotrypsinogen system). Observation of a similar N-terminal sequence in mature bacterial factors, which were known to activate their cognate mammalian zymogens *without* requiring initial proteolysis (e.g., SC and the plasminogen activator, streptokinase) let Bode and Huber to a profound conceptual proposal. They suggested that these bacterial cofactors utilize their N-terminal sequences to allosterically induce active site formation in their host zymogens, thereby bypassing the need for the proteolytic cleavage of the canonical Arg^15^-Ile^16^ peptide bond. This concept established the foundation for understanding cofactor-induced zymogen activation.

**Figure 2:**
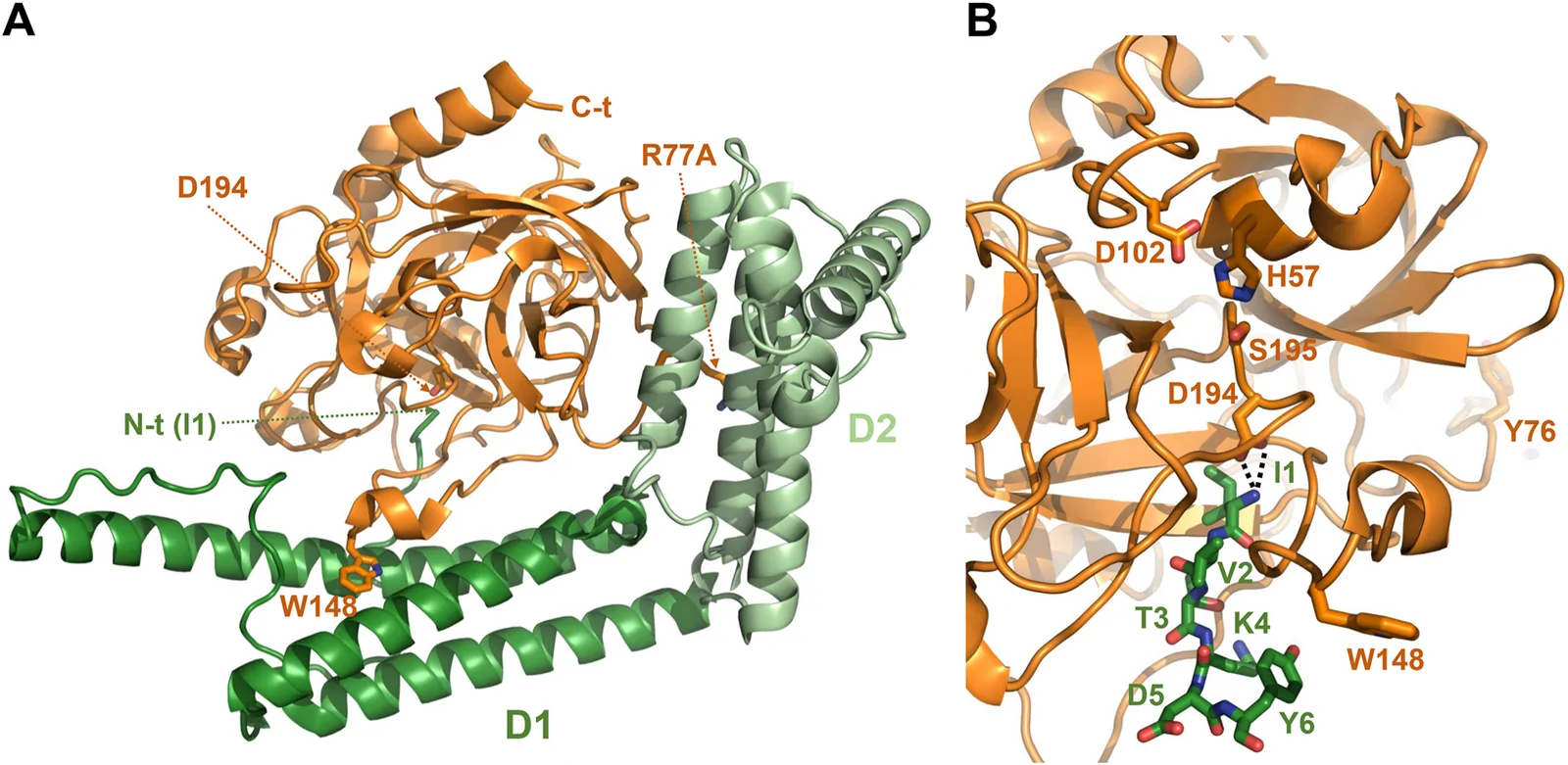
Structural basis of SC-mediated prothrombin activation. (A) Crystal structure of the SC·prethrombin-2 complex (PDB 1NU9). The α-helical domains D1 (forest green) and D2 (pale green) interact with the 148-loop and exosite I of the cognate thrombin zymogen (orange). See Supplementary Figure S2 for details of these interactions. (B) Closeup of the N-terminal SC activator peptide bound inside the Ile^16^-pocket of prethrombin-2. The six N-terminal residues of the bacterial ProT activator are shown as color-coded sticks and labeled.

Our X-ray crystal structure of the zymogen prethrombin-2 complexed with the SC(1–325) fragment provided the first structural proof of the mechanism of cofactor-induced zymogen activation. This structure revealed that the N-terminal SC peptide (Ile^1^-Val-Thr-Lys^4^) inserts deeply into the main catalytic domain (Friedrich et al. 2003) (Figure 2A). This insertion is stabilized by strong hydrogen-bonded salt bridges with the Asp^194^ carboxylate (Figure 2B), inducing a proteolytically competent conformation in the bound ProT that is structurally and functionally indistinguishable from free α-thrombin. Subsequent detailed fluorescence spectroscopy studies confirmed the stringent requirements for this interaction: extending the N-terminus by a single Met residue reduced ProT affinity 60-fold. Truncation of the Ile^1^ residue was deleterious (~six-fold reduction in affinity), and deletion of the first two residues essentially abolished activity (Friedrich et al. 2003). Finally, our thorough analysis of the contribution of these first two N-terminal residues to ProT binding and activation demonstrated the need for small aliphatic residues at these positions, leading to the identification of nearly-as-active SC variants (Val^1^/Val^2^, Ile^1^/Ala^2^ and Leu^1^/Val^2^) (Maddur et al. 2020). (SC numbering refers to the Tager 104 strain (Davis et al. 2016), but results are broadly extrapolated to all SC variants).

The N-terminal activator region of SC comprises two α-helical domains, D1 and D2, joined by a a short, well-defined linker. They contact each other through a small interface, which suggests considerable interdomain flexibility in solution - a feature that may be critical for complex formation with and activation of cognate ProT. Domain D1 binds the prominent 148- (or autolysis) loop, located to the “south” in Wolfram’s beloved standard orientation for proteinases (Figures 2A and S2A). This interaction precisely positions the activation peptide at the appropriate distance and orientation for entering the Ile^16^-pocket of bound ProT. The 148-loop builds the “gate” to the Ile^16^-pocket and is particularly relevant for SC’s specificity. Notably, the bulky basic side chains of Arg^144^ and Arg^145^ in bovine ProT might deflect the N-terminal SC peptide, which would explain why SC fails to activate the bovine zymogen, even though it binds α-thrombin from both species with similar affinity (Friedrich et al. 2006). (Human ProT features Leu and Lys at these positions, respectively). Domain D2 docks onto to a basic surface located to the “east”, formed by the 37- and 70–80 loops, and termed anion-binding exosite I (ABE-I), with important consequences for the mechanism of Fbg processing (see below) (Figures 2A and S2B). We have leveraged this detailed structural and functional information on SC-mediated ProT to develop fluorescent probes that allow for the highly sensitive, non-invasive detection of *S. aureus* infections (Panizzi et al. 2011).

### 2.2 The C-terminal region of staphylocoagulase presents a platform for high-affinity fibrinogen recognition

The Fbg molecule is a large, elongated glycoprotein composed of two identical pairs of Aα, Bβ, and γ chains, denoted as (Aα)_2_, (Bβ)_2_, *γ*_2_). These six chains are linked by multiple inter-chain disulfide bonds into a three-nodular structure. A central module, fragment E (Frag E), contains the N-termini of all six subunits and is connected by long coiled-coil segments to two peripheral D modules (Frag D). The E module harbours two pairs of acidic fibrinopeptides (FpA and FpB) at the N-termini of the Aα and Bβ chains, respectively. Their removal by α-thrombin is the key step in converting Fbg into clot-forming Fbn, allowing the resulting monomers to spontaneously polymerize by inserting the newly exposed *α* and *β* “knobs” into complementary “holes” (a and b) at the C-terminal ends of the *γ* and *β* chains, respectively (reviewed in Litvinov et al. 2005; Doolittle 2017; Wolberg 2023).

The crystal structures of α-thrombin bound to FpA (Stubbs et al. 1992) and Frag E (Pechik et al. 2004) established the structural basis for understanding fibrinogen recognition and processing. This structural research, combined with detailed functional studies, demonstrated that Fbg conversion to Fbn strictly depends on interactions with thrombin’s ABEI (Bock et al. 2007). Not surprisingly, this exosite is also targeted by potent inhibitors from various hematophagous animals (Corral-Rodríguez et al. 2009; Figueiredo et al. 2012; Macedo-Ribeiro et al. 2008; van de Locht et al. 1995). The essential role of exosite I is best illustrated by triabin, which docks exclusively at this site without occluding thrombin’s active center (Fuentes-Prior et al. 1997). Consequently, the thrombin·triabin complex retains activity against small substrates but is entirely incapable of activating Fbg (Noeske-Jungblut et al. 1995). In the light of this structural and functional evidence, the puzzling observation that the SC’s D2 domain blocks exosite I implies that the bacterial activator must provide an autonomous Fbg-recognition platform to ensure the rapid, specific cleavage of the only physiological substrate of SC· (Pro)T* complexes (Panizzi et al. 2006).

We and others have conclusively demonstrated that the C-terminal repeat domains of the SC molecule harbor the primary Fbg-recognition activity (Panizzi et al. 2011). Early deletion studies established that constructs lacking the C-terminal region fail to bind Fbg, whereas this fragment alone retains full binding activity (McDevitt et al. 1992). Importantly, quantitative binding analyses confirmed that the C-terminal half of the SC molecule possesses a 200-fold higher affinity for Fbg than the N-terminal domains (*K*_*D*_ = 4.6 × 10^−10^ vs. 2.0 × 10^−8^ M) (Thomas et al. 2019). Furthermore, published results imply the presence of two independent Fbg binding sites within this C-terminal region: the first is comprised of RD1, while a second, distinct site is contained within the remaining SC sequence (Bertoglio et al. 2023; Ko et al. 2016; Maddur et al. 2022; Thomas et al. 2019). Fibrinogen’s Frag D has been unambiguously identified as the target domain for the C-terminal repeats of SC (Maddur et al. 2022).

### 2.3 The C-terminal repeat domains of staphylocoagulase fold into two distinct, all-β modules

Despite extensive efforts, we have been unable to grow crystals of the C-terminal SC region, either free or complexed with its cognate Frag D. Circular dichroism and NMR results suggest this isolated fragment is largely intrinsically disordered in the absence of a ligand (Maddur et al. 2022; Voehler et al. 2017). Fortunately, the advent of high-accuracy protein structure prediction algorithms, spearheaded by the AlphaFold2 (AF2) revolution (Jumper et al. 2021), has provided new avenues for accurately modeling this elusive SC fragment. We have systematically employed currently available 3D structure prediction tools to develop models of the C-terminal SC domains. Remarkably, all algorithms consistently predict an all-β structure for this region and essentially identify the same residues as part of β-strands. (Figure S1 provides a structure-based sequence alignment of the C-terminal SC repeated sequences from representative staphylococcal strains). Furthermore, these programs coincidentally predict a three-stranded structure for the small RD1 domain.

**Figure 3:**
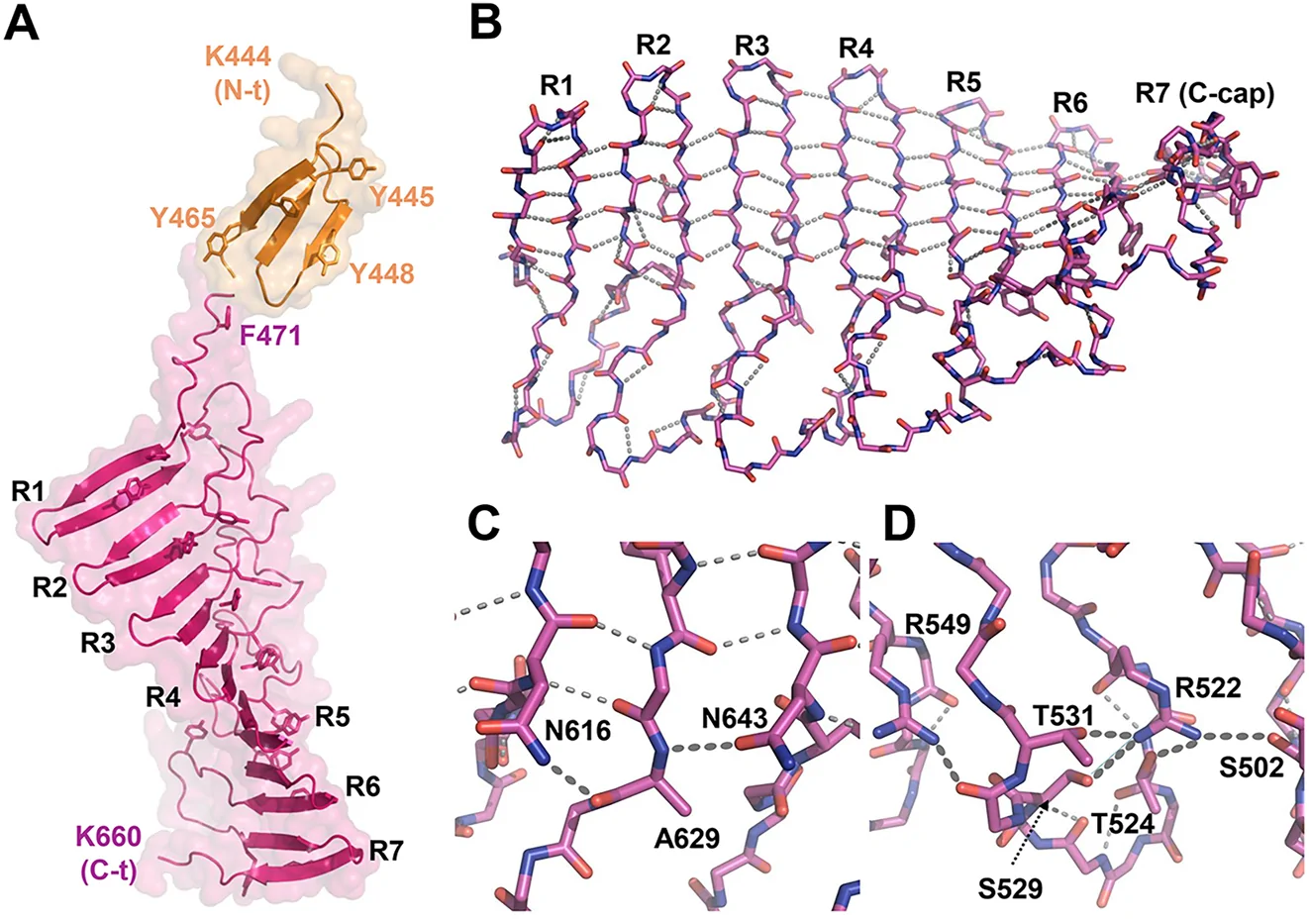
SC-RD2 folds into a single-chain, highly twisted β-sheet. (A) 3D model of the SC repeat domains. Domains RD1 (orange) and RD2 (hot pink) are shown as semitransparent surfaces with superimposed cartoons. Repeats are numbered R1-R7. The two modules are connected by a short linker and do not interact directly, suggesting they behave independently in solution – consistent with current immunological evidence. Tyr side chains are shown for comparison with alternative models. (B) Intra-main chain hydrogen bonding network in SC-RD2. H-bonds are depicted as gray dotted lines, highlighting the continuous network connecting neighboring repeats and the highly twisted character of this SLB. For clarity, only the aromatic Tyr side chains are shown. (C) The strictly conserved Asn at position +6 contributes to the stability of the domain. The panel illustrates Asn^616^ and Asn^643^ forming hydrogen bonds with the main chain O and N atoms of Ala^629^, respectively, in the β-strand “sandwiched” between the asparagine-bearing strands. (D) Stabilizing role of conserved Arg residues. The strictly conserved Arg residues at the start of the inter-repeat turns bridge the space between consecutive loops. As shown for Arg^522^, these residues donate stabilizing H-bonds to main chain carbonyls and/or Ser/Thr hydroxyl groups; while specific partners vary between repeats, the structural role remains consistent.

However, the generated models differ significantly in how the remaining strands are folded into the final 3D structure of the RD2 module. For example, most algorithms – including the AF2 models currently linked to SC entries in the UniProt database – predict a β-sandwich arrangement, with the RD1 module docked on one side as an N-terminal cap (Figures S3A and B). In contrast, other algorithms such as RoseTTAFold2 (Krishna et al. 2024) predict a barrel-like assembly of the RD2 repeats, which are distributed around a central open space (Figure S3C and D); comparable models are postulated for the prevalent SC variants characterized by six RD2 repeats. We currently favor an alternative model, generated by ColabFold (Kim et al. 2025; Mirdita et al. 2022), in which RD1 and RD2 modules do not directly contact each other. Notably, and in striking contrast to the sandwich and barrel structures, the β-strands in this RD2 model are arranged into a single-layer β-sheet (SLB) (Figure 3A). Consequently, SC-RD2 can be assigned to class 3.4.2 in the RepeatsDB database of Structured Tandem Repeat Proteins (https://repeatsdb.org/; Clementel et al. 2025).

Several lines of evidence suggest that the SLB arrangement represents the preferred, most stable conformation for the folded RD2 module. The architecture of another virulence factor, extracellular fibrinogen-binding protein (Efb), provides a key precedent: its N-terminal Fbg-binding moiety is built from two independent copies of the RD1 domain (termed Efb-A and Efb-O) (Figure 1). This modularity strongly suggests that the small RD1 domain can fold autonomously. Indeed, recombinant Efb fragments containing these individual domains exhibit distinct Fbg-binding activities (Bertoglio et al. 2023; Ko et al. 2016; Palma et al. 2001). Furthermore, the alternative β-sandwich model suffers from packing inefficiencies, particularly the poor stabilization of several tyrosine residues within its hydrophobic core (Figure S3B). Similarly, the open β-barrel arrangement relies on less robust stabilization, specifically hydrogen bonds formed between the hydroxyl groups of Tyr residues from one repeat and a main-chain oxygen atom from a neighboring repeat (Figure S3D). In striking contrast, the SLB is stabilized by an extensive network of inter-main chain H-bonds (Figure 3B), further supported by numerous side chain-to-main-chain and inter-side-chain H-bonds involving conserved polar/charged residues. Two of these interactions are particularly noteworthy, involving strictly conserved asparagine residues at position +6 (Figure 3C) and arginines at the start of the inter-repeat turn (position +20) (Figure 3D).

Nevertheless, the β-sandwich and open barrel conformations might represent alternative, less-populated states or folding intermediates of the RD2 domain that could still be relevant for *in vivo* Fbg recognition and processing. The divergence among the three prediction algorithms highlights the inherent bias of these computational tools; the models reflect the architectures each algorithm was trained to recognize. The fact that these predictions conflict with one another – and with our experimental CD and NMR data – suggests that RD2 exists as an intrinsically disordered region (IDR) or a highly dynamic ensemble, which may only adopt a stable structure upon binding to its partner, Fbg (see below).

### 2.4 SC-RD2 belongs to the family of MORN domain-containing proteins

Important, albeit indirect, support for the SLB model of the RD2 module comes from the significant structural similarity observed with the ubiquitously expressed tandem repeat proteins termed “Membrane Occupation and Recognition Nexus” (MORN) (InterPro IPR003409; https://www.ebi.ac.uk/interpro/entry/InterPro/IPR003409; reviewed in Zhou et al. 2022). Indeed, systematic searches using FoldSeek (van Kempen et al. 2024) revealed strong, previously unnoticed similarities between the SC SLB and a large number of MORN proteins (Supplementary Table 1 summarizes the closest structurally characterized RD2 homologs). These structural homologs are critically involved in the formation of highly specialized, stable macromolecular complexes that perform essential mechanical or organizational roles in the cell. Prominent examples include mammalian junctophilins (Yang et al. 2022), centromere proteins such as *Danio rerio* CenpJ/CPAP and its *Drosophila* ortholog, SAS-4 (Cottee et al. 2013; Hatzopoulos et al. 2013), and flagellar proteins recently characterized by cryo-EM (Grossman-Haham et al. 2021; Gui et al. 2022; Walton et al. 2023; Zheng et al. 2021).

**Figure 4:**
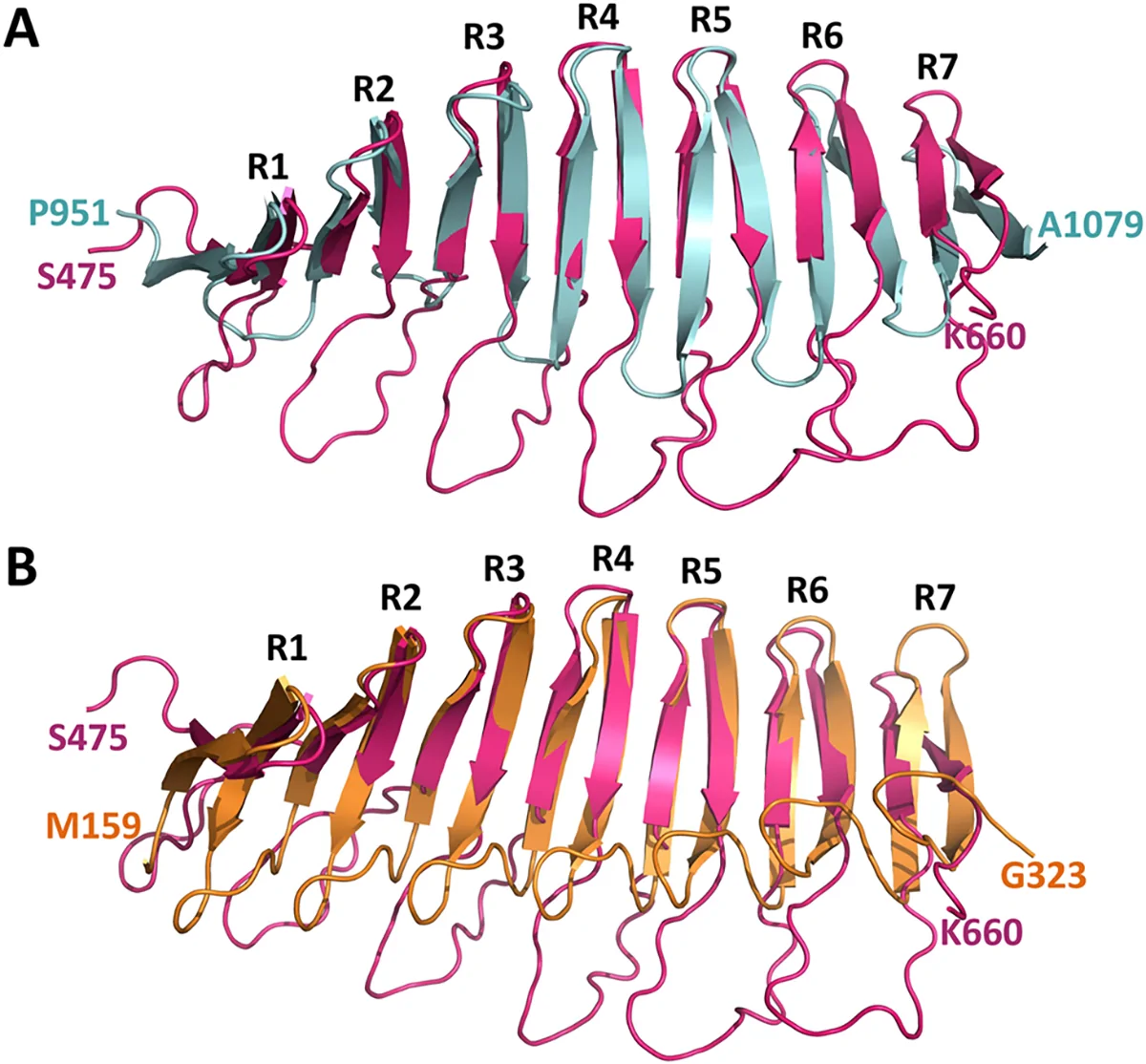
Structural homology between the SC-RD2 domain and the MORN superfamily. The SC-RD2 model (hot pink cartoon) is superimposed on the crystal structures of MORN domains from *Danio rerio* CenpJ/CPAP (aquamarine) (A) and *Toxoplasma gondii* MORN1 (orange) (B). For clarity, only the domain fragments structurally overlapping with SC-RD2 are displayed. The seven SC repeats are labeled R1-R7. Note the high degree of structural superimposition between the β-hairpins, particularly within the central repeats R3-R5. In contrast, the inter-repeat loops in SC are significantly longer and adopt a unique conformation compared to canonical MORN proteins. These proline-rigidified loops generate a more extended and structurally distinct surface in SC-RD2 than previously characterized members of the MORN superfamily. See Supplementary Figure S4 for sequence alignments.

This structural similarity extends to MORN proteins from three major human pathogens – *Toxoplasma gondii*, *Trypanosoma brucei* and *Plasmodium falciparum*; (Sajko et al. 2020) – as well as to OspA, the major surface antigen of the Lyme disease spirochete, *Borrelia burgdorferi* (Ding et al. 2000; Li et al. 1997; Schiller et al. 2021). The model of the SC SLB is shown superimposed on representative structures of CenpJ/CPAP and *T. gondii* MORN1 in Figure 4A and B, respectively. While SC and these MORN share a high degree of structural homology, sequence similarities lie within the “twilight zone” (typically 10–20 %; Figure S4). A defining structural distinction is that SC features significantly longer inter-repeat loops. FoldSeek identifies structural similarities in numerous structurally uncharacterized proteins across both procaryotes and eukaryotes. These include LPxTG domain-containing, cell wall-anchored proteins from various staphylococcal strains and the closely related *Mammaliicoccus sciuri* (formerly classified as *Staphylococcus sciuri*).

### 2.5 Implications for fibrinogen recognition and processing

**Figure 5:**
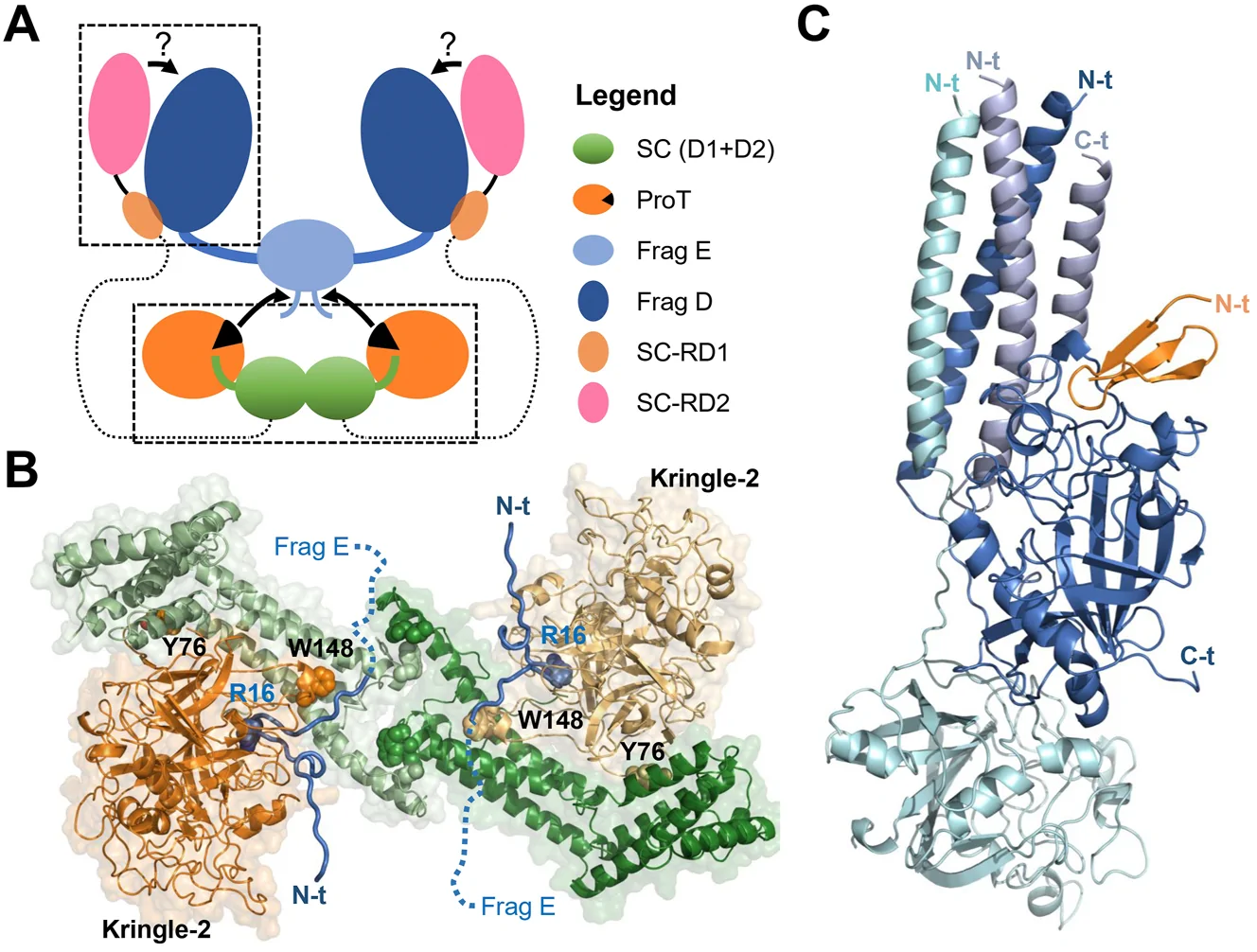
Mechanism of fibrinogen recognition and clotting by SC·ProT* complexes. (A) Schematic representation of the mechanism of Fbg recognition. The diagram highlights both crystallographically proven and modelled interactions. The long, unstructured linker connecting domains D2 and RD1 is omitted for clarity. Boxed areas correspond to the detailed interactions depicted in panels B and C. (B) D1-mediated SC·ProT* dimers. The SC·PreT1* complex – modelled based on the crystal structures of SC·PreT2* (1NU9; Friedrich et al. 2003) and ProT (4HZH; Pozzi et al. 2013) – is shown as a semi-transparent surface with superimposed cartoons. PreT1* molecules are coloured orange and light orange, while SC N-terminal domains are pale and forest green. Kringle 2 domains are labelled; notably, they are positioned on exosite II, and are thus located away from the SC binding surfaces on the catalytic domain. Aromatic residues at the interdigitating D1-D1 interface are depicted as spheres. Significant interface areas (615–620 Å^2^) and favourable Δ*G* values (−6.8/−7.5 kcal/mol) suggest that these dimers are biologically relevant. Accordingly, the Δ^*i*^*G P*-values (0.21/0.20) are comparable to or lower than those for the primary SC-(pre)thrombin interfaces. Side chains of residues Trp^148^ (interface with D1) and Tyr^76^ (interface with D2) are also shown as spheres for orientation. FpA peptides (blue ribbons) are modelled into both PreT1* active sites based on the α-thrombin–FpA complex structure (1FPH; Stubbs et al. 1992), with Arg^16^ residues shown as spheres. This dimeric arrangement could simultaneously accommodate and process both fibrinopeptides from a single Fbg molecule, as indicated. (C) Predicted model of SC-RD1 bound to the peripheral fragment D. Fbg *α*, *β* and *γ* chains are colored light blue, marine, and aquamarine, respectively. All generated models consistently place the RD1 modules within hole ‘b’ of the fibrinogen Frag D.

Currently available structural and functional evidence, integrated with state-of-the-art AI-derived modelling, allows for a unified model of fibrinogen clotting by SC·ProT* complexes *in vivo* (summarized in Figure 5A). First, our crystal structures of SC·(Pre)T* complexes reveal symmetric, anti-parallel dimers, formed through the interdigitation of the D1 domains (Friedrich et al. 2003; Friedrich et al. 2006) (Figure 5B). PISA analysis confirms the stability and biological relevance of this interface, suggesting that the crystallographic SC dimers are not mere packing artifacts. Indeed, we previously demonstrated that a single Fbg molecule binds with nanomolar affinity to two copies of the complex formed by the SC N-terminal domains and active-site-inhibited α-thrombin (Panizzi et al. 2006). These structural and biochemical data support a model of a pentameric (SC)_2_·(ProT*)_2_·Fbg Michaelis complex. In this assembly, the active sites of both SC-bound ProT* molecules are positioned to simultaneously engage and cleave the FpA peptides from the bound substrate.

Deriving accurate models of fibrinogen complexes with the RD1 and RD2 modules remains challenging, as the precise interacting residues have not been fully characterized. Nevertheless, combined evidence from *in vitro* studies of Fbg recognition by SC and Efb, alongside detailed structural information on the Fbg molecule, allows us to advance preliminary models to guide further mechanistic studies. Previous results indicate that the small RD1 module contains the minimal Fbg-binding motif, which interacts specifically with Fbg Frag D (Bertoglio et al. 2023; Maddur et al. 2022). Our preliminary modeling suggests that SC-RD1 docks onto hole ‘b’ of the Fbg fragment (Figure 5C). However, in striking contrast to the physiological interactions mediated by GPR (A) and GHRP (B) fibrin knobs, SC’s RD1 does not rely on basic Arg/Lys for Fbg recognition. Instead, hydrophobic and aromatic interactions predominate at the predicted interface. This model is strongly supported by our alanine scanning results, which demonstrated that these predicted contact residues are essential for binding (Maddur et al. 2022). Furthermore, the model offers an elegant explanation for the much larger affinity of Efb-O for Fbg compared to the N-terminal domain, Efb-A (Ko et al. 2011) (Figure S5). The predominantly hydrophobic character of the RD1–Frag D interface is further corroborated by the remarkably slow off-rate observed for the interaction, characteristic of a highly stable complex (Ko et al. 2011).

Fbg interactions with RD2 appear more intricate, perhaps reflecting the intrinsic propensity of these repeats to adopt distinct, interchangeable conformations in solution. Structural studies of various MORN proteins consistently show that the concave face of the β-sheet is ideally suited to accommodate peptides – from the same protein or binding partners – typically in α-helical conformations (see e.g., Li et al. 2019; Yang et al. 2022). Although this binding mode offers an appealing possibility for the docking of the N-terminal triple helix of Frag D, it contrasts with our functional findings. Specifically, our data demonstrate that the C-terminal SC repeats can simultaneously engage up to five Frag D molecules, with the individual repeats increasing the affinity of the RD1 domain for the Fbg module by one order of magnitude (from *K*_D_ ~50–130 nM to 7–32 nM; Maddur et al. 2022). Furthermore, Thomer and colleagues previously reported high-stoichiometry binding, with 3–4 mol of Fbg bound per mol of the SC C-terminal region (Thomer et al. 2016). This multi-site binding capability suggests that the RD2 module utilizes a distinct mechanism allowing for the simultaneous, high-affinity capture of multiple Fbg molecules, rather than a single interaction along the concave β-sheet face.

Unguided 3D structure predictions of the SC-RD2·Frag D complex consistently generate solutions in which the SC domain adopts an extended conformation. In these models, strictly conserved Arg or Lys residues within the inter-repeat loops insert into the highly acidic holes ‘a’ or ‘b’ of fibrinogen Frag D (Figure S6). These extended arrangements likely prevail in solution, particularly during initial SC-Fbg encounters. This structural interpretation provides an elegant explanation for our puzzling observation: full-length SC clotted fibrinogen with a lag phase and less efficiently than the truncated variant, SC(1–325), which lacks the high-affinity Fbg-binding functions of the repeated domains (Maddur et al. 2022). This disparity implies that the C-terminal RD2 domain sequesters the Fbg molecules in a non-productive conformation, likely by competing with RD1 for Fbg binding, rather than facilitating optimal substrate presentation for cleavage (Maddur et al. 2022). Thus, the repeated domains of staphylocoagulase, and especially RD2, may be more critical for secondary interactions within the final fibrin mesh than for the initial fibrinopeptide cleavage. These SC-Fbn interactions are hypothesized to generate thrombi distinctly different from those formed by α-thrombin. Specifically, SC-induced thrombi exhibit increased mechanical instability and heightened susceptibility to tPA cleavage, with important clinical consequences for septic embolism resulting from the rupture of endocardial vegetations (Farkas et al. 2019).

## 3 Conclusions and outlook

The allosteric prothrombin (ProT) activator, staphylocoagulase (SC), remains a paradigm for *S*. *aureus* virulence factors. While the mechanism of non-proteolytic ProT activation – mediated by the insertion of the SC N-terminal peptide into the Ile^16^-pocket and stabilized by the α-helical D1 and D2 domains – is well-documented, the role of the C-terminal repeat domains in substrate processing has remained elusive. Our current modeling experiments, integrated with previous *in vitro* and immunological evidence, address this gap by advancing testable 3D models of the SC repeat domains and their complexes with fibrinogen (Fbg). Our structural modeling suggests that SC’s C-terminal region folds into two distinct, essentially independent all-β domains. The smaller domain, RD1, is a three-strand module closely related to Efb, while the larger RD2 domain exhibits more complex folding behavior. We identify its most likely stable conformation as a single-layer β-sheet (SLB), highlighting a striking similarity to both eukaryotic and prokaryotic MORN repeat proteins. Furthermore, our model of the RD1 domain docked onto hole ‘b’ of the Fbg fragment D rationalizes our prior mutagenesis studies and provides a structural basis for the shared binding strategies of SC and Efb interaction. These models offer a platform for future verification via site-directed mutagenesis of predicted interface residues.

A deeper understanding of *S. aureus* pathogenesis and the identification of potential therapeutic targets is urgently needed given the growing global threat posed by MRSA. SC is a promising vaccine candidate (Missiakas and Schneewind 2016), with evidence from mice models demonstrating that it induces cross-reactive antibodies that protect against lethal bacteremia (Cheng et al. 2010; McAdow et al. 2012). Notably, antibodies raised against the C-terminal RDs elicit phagocytic killing of staphylococci and provide cross-protection across multiple MRSA isolates (Thomer et al. 2016). While the RD2 domain exhibits lower immunogenicity (Bertoglio et al. 2023), innovative strategies – such as fusion with the heavy chain of tetanus neurotoxin – have successfully generated robust protective responses (Qian et al. 2019). By providing a structural map of these domains, our work facilitates the rational design of next-generation vaccines, allowing for the targeted enhancement of immunogenic epitopes or the blockage of specific Fbg-binding interfaces.

Finally, our results underscore both the transformative power and the inherent limitations of AI-based structure prediction. While high confidence, “real looking” models can now be rapidly generated for practically any amino acid sequence, they must be treated as starting hypotheses for the rigorous design and interpretation of subsequent experiments. High-resolution experimental determination of proteins and protein complexes remains an essential and irreplaceable field of research, ensuring the lasting relevance of Wolfram’s legacy in defining the intricate relationship between protein structure and function.

## Supplementary Material

Supplementary material

**Supplementary Material:** This article contains supplementary material (https://doi.org/10.1515/hsz-2025-0253).

## Data Availability

All 3D models are available from the corresponding author (P.F.-P.) upon reasonable request.
