# Supplementary material for: Structural basis for the procoagulant activity of Staphylococcus aureus staphylocoagulase
Source: Biol Chem. Author manuscript; Available in PMC 2026 Sep 24. (PMC13605537; doi:10.1515/hsz-2025-0253)
Supplement: Supplementary file 1 [file NIHMS2206660-supplement-Supplementary_material.pdf]

Supplemental Information to

Structural basis for the procoagulant activity of *Staphylococcus aureus* staphylocoagulase

Pablo Fuentes-Prior,[1*] Ashoka A. Maddur,[2] Peter Panizzi,[3] David Gailani,[4] and Ingrid M. Verhamme[5*]

Corresponding authors:
Pablo Fuentes-Prior, Department of Biochemistry and Molecular Biomedicine, Faculty of Biology, University of Barcelona (UB), 08028 Barcelona, Spain
Email: fuentespriorpablo@gmail.com

Ingrid M Verhamme, Department of Pathology, Microbiology and Immunology, Vanderbilt University Medical Center, Nashville, TN, USA
Email: Ingrid.verhamme@vanderbilt.edu

```
                      .   450     .   460     .   470     .   480     .   490     .   500
SC_JCSC 7639          KYVKYRDAGTGIREYNDGTFGYEARPRFNKPS----------------------------       R1

SC_UC723              KYVKYRDAGTGIREYNDGTFGYEARPRFNKPSETNAYNVTTNQDGTVSYGARPTQNKPS-       R2

SC_AIS2002058         KYVKYRDAGTGIREYNDGTFGYEARPRFNKPSETNAYNVTTNQDGTVSYGARPTQNKPSK       R3
SC_JCSC 6688          KYVKYRDAGTGIREYNDGTFGYEARPRFNKSSETNAYNVTTNQDGTVTYGARPTQNKPSK       R3

SC_AIS2002055         KYVKYRDAGTGIREYNDGTFGYEARPRFNKPSETNAYNVTTHANGQVSYGARPTQNKPSK       R4
SC_UC18               KYVKYRDAGTGIREYNDGTFGYEARPRFNKPSETNAYNVTTNQDGTVSYGARPTQNKPSE       R4
SC_AIS2002054         KYVKYRDAGTGIREYNDGTFGYEARPRFNKPSETNAYNVTTNQDGTVSYGARPTQNKPSE       R4
SC_UB637              KYVKYRDAGTGIREYNDGTFGYEARPRFNKPSETNAYNVTTNQDGTVTYGARPTQKKPSK       R4
SC_LQ41               KYVKYRDAGTGIREYNDGTFGYEARPRFNKPSETNAYNVTTHANGQVSYGARPTQNKPSE       R4
SC_UC304              KYVKYRDAGTGIREYNDGTFGYEARPRFNKSSETNAYNVTTNQDGTVTYGARPTQNKPSK       R4

SC_NCTC 8325          KYVKYRDAGTGIREYNDGTFGYEARPRFNKPSETNAYNVTTHANGQVSYGARPTYKKPSE       R5
SC_IFH556             KYVKYRDAGTGIREYNDGTFGYEARPRFNKPSETNAYNVTTNQDGTVSYGARPTQNKPSK       R5
SC_MW2                KYVKYRDAGTGIREYNDGTFGYEARPRFNKPSETNAYNVTTNQDGTVTYGARPTQNKPSK       R5
SC_MMR-V              KYVKYRDAGTGIREYNDGTFGYEARPRFNKPSETNAYNVTTNQDGTVTYGARPTQNKPSK       R5
SC_19                 KYVKYRDAGTGIREYNDGTFGYEARPRFNKPSETNAYNVTTNQDGTVTYGARPTQKKPSK       R5
SC_UCL341             KYVKYRDAGTGIREYNDGTFGYEARPRFNKPSETNAYNVTTNQDGTVTYGARPTQNKPSK       R5
SC_SH751              KYVKYRDAGTGIREYNDGTFGYEARPRFNKPSETNAYNVTTHGNGQVSYGARPTQNKPSK       R5
SC_JCSC 7623          KYVKYRDAGTGIREYNDGTFGYEARPRFNKPSETNAYNVTTNQDGTVTYGARPTQNKPSK       R5
SC_Dresden-275757     KYVKYRDAGTGIREYNDGTFGYEARPRFNKPSETNAYNVTTNQDGTVTYGARPTQNKPSK       R5
SC_S_2023.LVRQ.AN     KYVKYRDAGTGIREYNDGTFGYEARPRFNKPSETNAYNVTTNQDGTVSYGARPTQKKPSE       R5
SC_UE1109             KYVKYRDAGTGIREYNDGTFGYEARPRFNKPSETNAYNVTTNQDGTVTYGARPTQNKASK       R5
SC_UE1097             KYVKYRDAGTGIREYNDGTFGYEARPRFNKPSETNAYNVTTNQDGTVSYGARPTQNKPSE       R5

SC_213                KYVKYRDAGTGIREYNDGTFGYEARPRFNKPSETNAYNVTTNQDGTVSYGARPTQNKPSE       R6
SC_SG03               KYVKYRDAGTGIREYNDGTFGYEARPRFNKPSETNAYNVTKHANGQVTYGARPVQNKPSK       R6
SC_Stp-12             KYVKYRDAGTGIREYNDGTFGYEARPRFNKPSETNAYNVTTNQDGTVTYGARPTQNKASK       R6
SC_SH60               KYVKYRDAGTGIREYNDGTFGYEARPRFNKPSETNAYNVTTNQDGTVTYGARPTQNKASK       R6
SC_JCSC6074           KYVKYRDAGTGIREYNDGTFGYEARPRFNKPSETNAYNVTTNQDGTVSYGARPTQNKPSK       R6
SC_JCSC1469           KYVKYRDAGTGIREYNDGTFGYEARPRFNKPSETNAYNVTTHANGQVTYGARPTQNKPSK       R6
SC_JCSC6075           KYVKYRDAGTGIREYNDGTFGYEARPRFNKPSETNAYNVTTNQDGTVTYGARPTQNKPSK       R6
SC_JCSC6669           KYVKYRDAGTGIREYNDGTFGYEARPRFNKPSETNAYNVTTNQDGTVSYGARPTYKKPSE       R6
SC_AH1                KYVKYRDAGTGIREYNDGTFGYEARPRFNKPSETNAYNVTTNQDGTVSYGARPTQKKPSE       R6
SC_67-013             KYVKYRDAGTGIREYNDGTFGYEARPRFNKPSETNAYNVTTNQDGTVTYGARPTQNKPSK       R6
SC_SH640              KYVKYRDAGTGIREYNDGTFGYEARPRFNKPSETNAYNVTTNQDGTVTYGARPTQNKPSK       R6
SC_01093              KYVKYRDAGTGIREYNDGTFGYEARPRFNKPSETNAYNVTTNQDGTVTYGARPTQNKPSK       R6
SC_IRQBAScoa28        KYVKYRDAGTGIREYNDGTFGYEARPRFNKPSETNAYNVTTNQDGTVSYGARPTQNKPSE       R6
SC_JCSC6665           KYVKYRDAGTGIREYNDGTFGYEARPRFNKPSETNAYNVTTNQDGTVSYGARPTQNKPSK       R6
SC_UE1112             KYVKYRDAGTGIREYNDGTFRYEARPRFNKPSETNAYNVTTHANGQVSYGARPTQNKPSK       R6
SC_17573              KYVKYRDAGTGIREYNDGTFRYEARPRFNKPSETNAYNVTTNQDGTVSYGARPTQNKPSE       R6
SC_85/2232            KYVKYRDAGTGIREYNDGTFGYEARPRFNKPSETNAYNVTTNQDGTVSYGARPTQNKPSE       R6
SC_SH488              KYVKYRDAGTGIREYNDGTFGYEARPRFNKPSETNAYNVTTNQDGTVTYGARPTQNKASK       R6

SC_Tager 104          KYVKYRDAGTGIREYNDGTFGYEARPRFNKPSETNAYNVTTHANGQVSYGARPTQNKPSK       R7
SC_NCTC13712          KYLKYRDAGTGIREYNDGTYGYEARPRFNKPSETNAYNVTTHANGQVSYGARPTQNKPSK       R7
SC_104                KYVKYRDAGTGIREYNDGTFGYEARPRFNKPSETNAYNVTTHANGQVSYGARPTQNKPSK       R7
SC_S_4031.LGMP.AI     KYVKYRDAGTGIREYNDGTFGYEARPRFNKPSETNAYNVTTNQDGTVSYGARPTQKKPSE       R7
SC_SH407              KYVKYRDAGTGIREYNDGTFGYEARPRFNKPSETNAYNVTTHANGQVSYGARPTQNKPSE       R7
SC_4074               KYVKYRDAGTGIREYNDGTFGYEARPRFNKPSETNAYNVTTNQDGTVTYGARPTQNKPSK       R7
SC_M9N                KYVKYRDAGTGIREYNDGTFGYEARPRFNKPSETNAYNVTTNQDGTVTYGARPTQNKPSK       R7
SC_JCSC6908           KYVKYRDAGTGIREYNDGTFGYEARPRFNKPSETNAYNVTTHANGQVSYGARPTQNKPSE       R7

SC_BB                 KYVKYRDAGTGIREYNDGTFGYEARPRFNKPSETNAYNVTTNQDGTVSYGARPTQNKASE       R8
SC_JCSC 7552          KYVKYRDAGTGIREYNDGTFGYEARPRFNKPSETNAYNVTTNQDGTVSYGARPTYNKPSE       R8
SC_JCSC 7619          KYVKYRDAGTGIREYNDGTFGYEARPRFNKPSETNAYNVTTNQDGTVSYGARPTQNKASE       R8
SC_IFH467             KYVKYRDAGTGIREYNDGTFGYEARPRFNKPSETNAYNVTTNQDGTVTYGARPTQNKPSE       R8
SC_Stp25              KYVKYRDAGTGIREYNDGTFGYEARPRFNKPSETNAYNVMTNQDGTVSYGARPTQNKASE       R8
SC_NCTC7972           KYVKYRDAGTGIREYNDGTFGYEARPRFNKPSETNAYNVTTNQDGTVSYGARPTQNKPSE       R8

SC_JCSC 7553          KYVKYRDAGTGIREYNDGTFGYEARPRFNKPSETNAYNVTTNQDGTVSYGARPTYNKPSE       R9
SC_Ku                 KYVKYRDAGTGIREYNDGTFGYEARPRFNKPSETNAYNVTTNQDGTVSYGARPTQNKPSE       R9
                      **:****************: *********:********     :* *:*****:   *:*
                      <----------- RD1 ------>< L ><----- R1 (N-t cap) ----><

                      KYVKYRDAGTGIREYNDGTFGYEARPRFNKPSETNAYNVTTNQDGTVSYGARPTQNKPSE
                        L                YR         S        MKHAN Q T     VYK A K
                                                                G
```

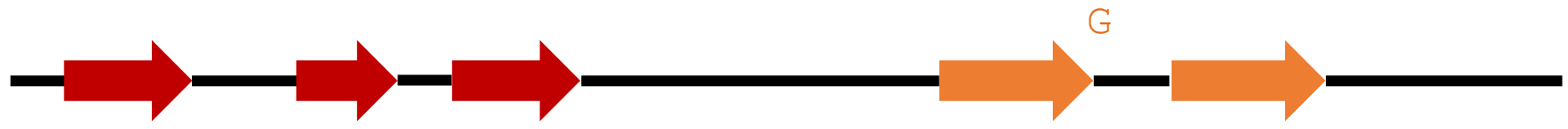

```
                           .  510    .  520    .  530    .  540    .  550    .  560
SC_JCSC 7639          -------------------------------------------------------------   R1
SC_UC723              -------------------------------------------------------------   R2
SC_AIS2002058         TNAYNVTTHANGQVSYGARPTYNKPS-----------------------------------   R3
SC_JCSC 6688          TNAYNVTTHANGQVSYGARPTQKKPS-----------------------------------   R3
SC_AIS2002055         TNAYNVTTHGNGQVSYGARPTQNKPSKTNAYNVTTHANGQVSYGARPTYKKPS-------   R4
SC_UC18               TNAYNVTTHANGQVSYGARPTYKKPSETNAYNVTTHANGQVSYGARPTQKKPS-------   R4
SC_AIS2002054         TNAYNVTTHANGQVSYGARPTQNKPSETNAYNVTTHANGQVSYGARPTQNKPS-------   R4
SC_UB637              TNAYNVTTHANGQVSYGARPTQKKPSKTNAYNVTTHANGQVSYGARPTYKKSS-------   R4
SC_LQ41               TNAYNVTTHANGQVSYGARPTQNKPSETNAYNVTTNQDGTVSYGARPTQNKPS-------   R4
SC_UC304              TNAYNVTTHANGQVSYGARPTQNKPSKTNAYNVTTHANGQVSYGARPTQKKPS-------   R4
SC_NCTC 8325          TNAYNVTTHANGQVSYGARPTQNKPSKTNAYNVTTHGNGQVSYGARPTQNKPSKTNAYNV   R5
SC_IFH556             TNAYNVTTHANGQVSYGARPTQNKPSKTNEYNVTTHANGQVSYGARPTYKKPSETNAYNV   R5
SC_MW2                TNAYNVTTHANGQVSYGARPTQNKPSKTNAYNVTTHANGQVSYGARPTQNKPSKTNAYNV   R5
SC_MMR-V              TNAYNVTTHANGQVSYGARPTQNKPSKTNAYNVTTHANGQVSYGARPTYKKPSETNAYNV   R5
SC_19                 TNAYNVTTHANGQVSYGARPTQKKPSKTNAYNVTTHANGQVSYGARPTYKKSSETNAYNV   R5
SC_UCL341             TNAYNVTTHGNGQVSYGARPTQNKPSKTNAYNVTTHANGQVSYGARPTQNKASETNAYNV   R5
SC_SH751              TNAYNVTTHANGQVSYGARPTQNKPSKTNAYNVTTHANGQVSYGARPTYKKPSETNAYNV   R5
SC_JCSC 7623          TNAYNVTTHANGQVSYGARPTQKKPSKTNAYNVTTHANGQVSYGARPTQKKPSKTNAYNV   R5
SC_Dresden-275757     TNAYNVTTHANGQVSYGARPTQKKPSKTNAYNVTTHANGQVSYGARPTQKKPSETNAYNV   R5
SC_S_2023.LVRQ.AN     TNAYNVTTHANGQVSYGARPTYNKPSKTNAYNVTTHANGQVSYGARPTQNKPSETNAYNV   R5
SC_UE1109             TNAYNVTTHANGQVSYGARPTYKKPSETNAYNVTTHANGQVSYGARPTQNKPSETNAYNV   R5
SC_UE1097             TNAYNVTTHANGQVSYGARPTQNKPSETNAYNVTTHANGQVSYGARPTQNKPSETNAYNV   R5
SC_213                TNAYNVTTHANGQVSYGARPTQNKPSKTNAYNVTTHANGQVSYGARPTQKKPSKTNAYNV   R6
SC_SG03               TNAYNVTTHANGQVSYGARPAQNKPSKTNAYNVTTHANGQVSYGARPAQNKPSKTNAYNV   R6
SC_Stp-12             TNAYNVTTHANGQVSYGARPTYKKPSKTNAYNVTTHANGQVSYGARPTQNKPSKTNAYNV   R6
SC_SH60               TNAYNVTTHANGQVSYGARPTYKKPSETNAYNVTTHANGQVSYGARPTQNKASETNAYNV   R6
SC_JCSC6074           TNAYNVTTHGNGQVSYGARPTYKKPSETNAYNVTTHANGQVSYGARPTYKKPSETNAYNV   R6
SC_JCSC1469           TNAYNVTTHANGQVSYGARPAQNKPSKTNAYNVTTHANGQVSYGARPAQNKPSKTNAYNV   R6
SC_JCSC6075           TNAYNVTTHANGQVSYGARPTYKKPSETNAYNVTTHANGQVSYGARPTQNKASETNAYNV   R6
SC_JCSC6669           TNAYNVTTHANGQVSYGARPTQNKPSETNAYNVTTHGNGQVSYGARPTQNKPSKTNAYNV   R6
SC_AH1                TNAYNVTTHANGQVSYGARPTYNKPSKTNAYNVTTHANGQVSYGARPTQNKPSETNAYNV   R6
SC_67-013             TNAYNVTTHANGQVSYGARPTQNKPSKTNAYNVTTHANGQVSYGARPTQNKPSKTNAYNV   R6
SC_SH640              TNAYNVTTHGNGQVSYGARPTQNKPSKTNAYNVTTHANGQVSYGARPTYKKPSETNAYNV   R6
SC_01093              TNAYNVTTHANGQVSYGARPTQNKPSKTNAYNVTTHANGQVSYGARPTQNKPSKTNAYNV   R6
SC_IRQBAScoa28        TNAYNVTTNQDGTVSYGARPTQNKPSETNAYNVTTHANGQVSYGARPTQNKPSKTNAYNV   R6
SC_JCSC6665           TNAYNVTTHANGQVSYGARPTYNKPSETNAYNVTTNRDGTVSYGARPTQNKPSETNAYNV   R6
SC_UE1112             TNAYNVTTHANGQVSYGARPTQNKPSKTNAYNVTTHGNGQVSYGARPTYNKPSKTNAYNV   R6
SC_17573              TNAYNVTTHGNGQVSYGARPTYKKPSKTNAYNVTTHANGQVSYGARPTHNKPSETNAYNV   R6
SC_85/2232            TNAYNVTTHANGQVSYGARPTYKKPSETNAYNVTTNQDGTVSYGARPTQNKPSETNAYNV   R6
SC_SH488              TNAYNVTTHANGQVSYGARPTYKKPSETNAYNVTTHANGQVSYGARPTQNKPSKTNAYNV   R6
SC_Tager 104          TNAYNVTTHGNGQVSYGARPTYKKPSKTNAYNVTTHANGQVSYGARPTQNKPSKTNAYNV   R7
SC_NCTC13712          TNAYNVTTHANGQVSYGARPTYKKASETNAYNVTTHTNGQVSYGARPTQNKPSETNAYNV   R7
SC_104                TNAYNVTTHGNGQVSYGARPTYKKPSKTNAYNVTTHANGQVSYGARPTQNKPSKTNAYNV   R7
SC_S_4031.LGMP.AI     TNAYNVTTHANGQVSYGARPTYNKPSKTNAYNVTTHANGQVSYGARPTQNKPSETNAYNV   R7
SC_SH407              TNAYNVTTHANGQVSYGARPTQNKPSETNAYNVTTNQDGTVSYGARPTQNKPSKTNAYNV   R7
SC_4074               TNAYNVTTNQDGTVTYGARPTYKKPSETNAYNVTTHANGQVSYGARPTYNKPSKTNAYNV   R7
SC_M9N                TNAYNVTTHANGQVSYGARPTYKKPSKTNAYNVTTHANGQVSYGARPTQNKPSKTNAYNV   R7
SC_JCSC6908           TNAYNVTTNQDGTVSYGARPTQNKPSKTNAYNVTTHANGQVSYGARPTYNKPSETNAYNV   R7
SC_BB                 TNAYNVTTHANGQVSYGARPTQKKPSETNAYNVTTHANGQVSYGARPTYNKPSETNAYNV   R8
SC_JCSC 7552          TNAYNVTTHANGQVSYGARPTYKKPSETNAYNVTTHANGTVSYGARPTYKKPSKTNAYNV   R8
SC_JCSC 7619          TNAYNVTTHANGQVSYGARPTQKKPSETNAYNVTTHANGQVSYGARPTYNKPSETNAYNV   R8
SC_IFH467             TNAYNVTTHANGQVSYGARPTYKKPSKTNAYNVTTNQDGTVSYGARPTQNKPSKTNAYNV   R8
SC_Stp25              TNAYNVTTHANGQVSYGARPTQKKPSETNAYNVTTHANGQVSYGARPTYKKPSETNAYNV   R8
SC_NCTC7972           TNAYNVTTHANGQVSYGARPTQNKPSETNAYNVTTHANGQVSYGARPSQNKPSETNAYNV   R8
SC_JCSC 7553          TNAYNVTTHGNGQVSYGARPTQNKPSETNAYNVTTHANGQVSYGARPTQNKPSKTNEYNV   R9
SC_Ku                 TNAYNVTTHANGQVSYGARPTYKKPSETNAYNVTTHANGQVSYGARPTQNKPSETNAYNV   R9
                      ********  :*:* *****:* * * ** *****  * *******:  * * ** ***
                      --------- R2 ----------→←----------- R3 ---------→←-- R4
                      TNAYNVTTHANGQVSYGARPTYKKPSETNAYNVTTHANGQVSYGARPTQNKPSETNAYNV
                              NQD T T     AQN A K  E       NQD T        AYK A K  E
                               G                            G           SH  S
                                                            R
```

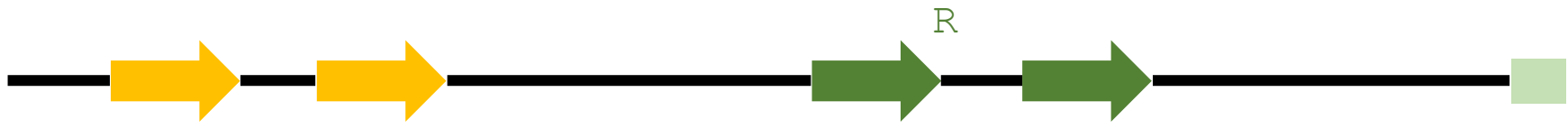

```
                     .  570    .  580    .  590    .  600    .  610    .  620
SC_JCSC 7639         ------------------------------------------------------------   R1
SC_UC723             ------------------------------------------------------------   R2
SC_AIS2002058        ------------------------------------------------------------   R3
SC_JCSC 6688         ------------------------------------------------------------   R3
SC_AIS2002055        ------------------------------------------------------------   R4
SC_UC18              ------------------------------------------------------------   R4
SC_AIS2002054        ------------------------------------------------------------   R4
SC_UB637             ------------------------------------------------------------   R4
SC_LQ41              ------------------------------------------------------------   R4
SC_UC304             ------------------------------------------------------------   R4
SC_NCTC 8325         TTHANGQVSYGARPTYKKPS----------------------------------------   R5
SC_IFH556            TTHANGQVSYGARPTQNKPS----------------------------------------   R5
SC_MW2               TTHANGQVSYGARPTYKKPS----------------------------------------   R5
SC_MMR-V             TTHANGQVSYGARPTQKKPS----------------------------------------   R5
SC_19                TTHANGQVSYGARPTYKKSS----------------------------------------   R5
SC_UCL341            TTHANGQVSYGARPTYKKPS----------------------------------------   R5
SC_SH751             TTHANGQVSYGARPTQKKPS----------------------------------------   R5
SC_JCSC 7623         TTHANGQVSYGARPTYKKSS----------------------------------------   R5
SC_Dresden-275757    TTHANGQVSYGARPTQKKPS----------------------------------------   R5
SC_S_2023.LVRQ.AN    TTHANGQVSYGARPTQNKPS----------------------------------------   R5
SC_UE1109            TTHANGQVSYGARPTYKKPS----------------------------------------   R5
SC_UE1097            TTHANGQVSYGARPTQNKPS----------------------------------------   R5
SC_213               TTHANGQVSYGARPTYKKPSETNAYNVTTHANGQVSYGARPTQKKPS-------------   R6
SC_SG03              TTHANGQVSYGARPAQNKPSKTNAYNVTTHANGQVTYGARPAQNKPS-------------   R6
SC_Stp-12            TTHANGQVSYGARPTYKKPSETNAYNVTTHANGQVSYGARPTQKKPS-------------   R6
SC_SH60              TTHANGQVSYGARPTQNKPSKTNAYNVTTHGNGQVSYGARPTYKKPS-------------   R6
SC_JCSC6074          TTHANGQVSYGARPTQNKPSKTNAYNVTTHANGQVSYGARPTYKKPS-------------   R6
SC_JCSC1469          TTHANGQVSYGARPAQNKPSKTNAYNVTTHANGQVTYGARPAQNKPS-------------   R6
SC_JCSC6075          TTHANGQVSYGARPTQNKPSKTNAYNVTTHGNGQVSYGARPTYKKPS-------------   R6
SC_JCSC6669          TTNQDGTVSYGARPTQNKPSETNAYNVTTHANGQVSYGARPTYNKPS-------------   R6
SC_AH1               TTHANGQVSYGARPTQNKPSETNAYNVTTHANGQVSYGARPTQNKPS-------------   R6
SC_67-013            TTHANGQVSYGARPTQNKPSKTNAYNVTTHANGQVSYGARPTYKKPS-------------   R6
SC_SH640             TTHANGQVSYGARPTQNKASETNAYNVTTHANGQVSYGARPTYKKPS-------------   R6
SC_01093             TTHANGQVSYGARPTYKKPSETNAYNVTTHANGQVSYGARPTQNKPS-------------   R6
SC_IRQBAScoa28       TTHANGQVSYGARPTQNKPSETNAYNVTTHANGQVSYGARPTQNKPS-------------   R6
SC_JCSC6665          TTHGNGQVSYGARPTQKKPSKTNAYNVTTHANGQVSYGARPTYNKPS-------------   R6
SC_UE1112            TTHANGQISYGARPTYKKPSETNAYNVTTHANGQVSYGARPTYNKPS-------------   R6
SC_17573             TTHGNGQVSYGARPTHNKPSETNAYNVTTHANGQVSYGARPTHNKPS-------------   R6
SC_85/2232           TTHANGQVSYGARPTQNKPSETNAYNVTTHANGQVSYGARPTQNKPS-------------   R6
SC_SH488             TTHANGQVSYGARPTQNKPSETNAYNVTTHANGQVSYGARPTYKKPS-------------   R6
SC_Tager 104         TTHGNGQVSYGARPTQNKPSKTNAYNVTTHGNGQVSYGARPTQNKPSKTNAYNVTTHANG   R7
SC_NCTC13712         TTHANGQVSYGARPAQNKPSKTNAYNVTTHANGQVSYGARPTYKKASETNAYNVTTHANG   R7
SC_104               TTHGNGQVSYGARPTQNKPSKTNAYNVTTHGNGQVSYGARPTQNKPSKTNAYNVTTHANG   R7
SC_S_4031.LGMP.AI    TTHANGQVSYGARPTQNKPSETNAYNVTTHANGQVSYGARPTQNKPSETNAYNVTTHANG   R7
SC_SH407             TTHANGQVSYGARPTQNKPSETNAYNVTTHANGQVSYGARPTQNKPSETNAYNVTTHGNG   R7
SC_4074              TTHANGQVSYGARPTYNKPSKTNAYNVTTHANGQVSYGARPTQNKPSETNAYNVTTHANG   R7
SC_M9N               TTHANGQVSYGARPTQNKPSETNAYNVTTHANGQVSYGARPTQNKPSETNAYNVTTHANG   R7
SC_JCSC6908          TTHANGQVSYGARPTYKKPSETNAYNVTTHANGQVSYGARPTQNKPSKTNAYNVTTHANG   R7
SC_BB                TTHGNGQVSYGARPTYKKPSKTNAYNVTTHANGQVSYGARPTQNKPSETNAYNVTTHANG   R8
SC_JCSC 7552         TTHANGQVSYGARPTQNKPSKTNAYNVTTHANGQVSYGARPTQNKPSKTNAYNVTTHGNG   R8
SC_JCSC 7619         TTHGNGQVSYGARPTYKKPSKTNAYNVTTHANGQVSYGARPTQKKPSETNAYNVTTHANG   R8
SC_IFH467            TTHANGQVSYGARPTYKKPSKTNAYNVTTHANGQVSYGARPTQKKPSKTNAYNVTTHANG   R8
SC_Stp25             TTHANGQVSYGARPTQNKPSNTNAYNVTTHANGQVSYGARPTQNKASETNAYNVTTHANG   R8
SC_NCTC7972          TTHANGQVSYGARPTYNKPSKTNAYNVTTHANGQVSYGARPTQNKPSETNAYNVTTHANG   R8
SC_JCSC 7553         TTHANGQVSYGARPTYKKPSETNAYNVTTHANGQVSYGARPTQNKPSETNAYNVTTHGNG   R9
SC_Ku                TTHANGQVSYGARPSQNKPSETNAYNVTTHANGQVSYGARPTYNKPSKTNAYNVTTHANG   R9
                     *** :**:******:   * * ********* ****:*****:   * * ********* **
                     ------- R4 ------->←----------- R5 --------->←------- R6
                     TTHANGQVSYGARPTQNKPSETNAYNVTTHANGQVSYGARPTYNKPSKTNAYNVTTHANG
                        GD I      SYK S K          G    T      AQK A E          G
                                  A   A                          H
```

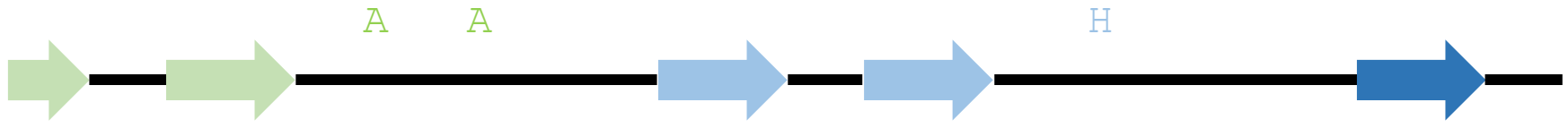

```
                           .    630    .
SC_JCSC 7639          ------------------------------------------------------------   R1

SC_UC723              ------------------------------------------------------------   R2

SC_AIS2002058         ------------------------------------------------------------   R3
SC_JCSC 6688          ------------------------------------------------------------   R3

SC_AIS2002055         ------------------------------------------------------------   R4
SC_UC18               ------------------------------------------------------------   R4
SC_AIS2002054         ------------------------------------------------------------   R4
SC_UB637              ------------------------------------------------------------   R4
SC_LQ41               ------------------------------------------------------------   R4
SC_UC304              ------------------------------------------------------------   R4

SC_NCTC 8325          ------------------------------------------------------------   R5
SC_IFH556             ------------------------------------------------------------   R5
SC_MW2                ------------------------------------------------------------   R5
SC_MMR-V              ------------------------------------------------------------   R5
SC_19                 ------------------------------------------------------------   R5
SC_UCL341             ------------------------------------------------------------   R5
SC_SH751              ------------------------------------------------------------   R5
SC_JCSC 7623          ------------------------------------------------------------   R5
SC_Dresden-275757     ------------------------------------------------------------   R5
SC_S_2023.LVRQ.AN     ------------------------------------------------------------   R5
SC_UE1109             ------------------------------------------------------------   R5
SC_UE1097             ------------------------------------------------------------   R5

SC_213                ------------------------------------------------------------   R6
SC_SG03               ------------------------------------------------------------   R6
SC_Stp-12             ------------------------------------------------------------   R6
SC_SH60               ------------------------------------------------------------   R6
SC_JCSC6074           ------------------------------------------------------------   R6
SC_JCSC1469           ------------------------------------------------------------   R6
SC_JCSC6075           ------------------------------------------------------------   R6
SC_JCSC6669           ------------------------------------------------------------   R6
SC_AH1                ------------------------------------------------------------   R6
SC_67-013             ------------------------------------------------------------   R6
SC_SH640              ------------------------------------------------------------   R6
SC_01093              ------------------------------------------------------------   R6
SC_IRQBAScoa28        ------------------------------------------------------------   R6
SC_JCSC6665           ------------------------------------------------------------   R6
SC_UE1112             ------------------------------------------------------------   R6
SC_17573              ------------------------------------------------------------   R6
SC_85/2232            ------------------------------------------------------------   R6
SC_SH488              ------------------------------------------------------------   R6

SC_Tager 104          QVSYGARPTYKKPS----------------------------------------------   R7
SC_NCTC13712          QVSYGARPTYKKAS----------------------------------------------   R7
SC_104                QVSYGARPTYKKPS----------------------------------------------   R7
SC_S_4031.LGMP.AI     QVSYGA RPTQNKPS----------------------------------------------  R7
SC_SH407              QVSYGARPTQNKPS----------------------------------------------   R7
SC_4074               QVSYGARPTQNKPS----------------------------------------------   R7
SC_M9N                QVSYGARPTQKKPS----------------------------------------------   R7
SC_JCSC6908           QVSYGARPTYKKPS----------------------------------------------   R7

SC_BB                 QVSYGARPTQNKPSETNAYNVTTHGNGQVSYGARPTYNKPS------------------   R8
SC_JCSC 7552          QVSYGARPTYKKPSETNAYNVTTHANGQVSYGARPTQNKPS------------------   R8
SC_JCSC 7619          QVSYGARPTQKKPSETNAYNVTTHGNGQVSYGARPTYNKPS------------------   R8
SC_IFH467             QVSYGARPTYKKPSETNAYNVTTHANGQVSYGARPTQKKPS-----------------    R8
SC_Stp25              QVSYGARPTQNKPSETNAYNVTTHGNGQVSYGARPTYNKPS------------------   R8
SC_NCTC7972           QVSYGARPTQNKPSETNAYNVTTHANGQVSYGARPTQNKPS------------------   R8

SC_JCSC 7553          QVSYGARPTQNKPSKTSAYNVTTHANGQVSYGARPTQNKPSKTNAYNVTTHANGQVSYGA   R9
SC_Ku                 QVSYGARPTQNKPSETNAYNVTTHANGQVSYGARPTQNKPSETNAYNVTTHANGQVSYGA   R9
                      *********  * * * ******* ***********  *** ******************
                      ---- R6 --->←----------- R7 --------->←---------- R8 ---
                      QVSYGARPTQNKPSETNAYNVTTHANGQVSYGARPTQNKPSETNAYNVTTHANGQVSYGA
                               YK A K S       G           YK   K
```

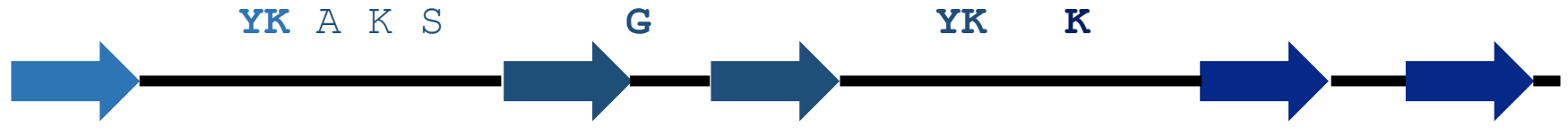

```
                                640    .  650    .  660
SC_JCSC 7639          --------ETNAYNVTTHADGTATYGPRVTK          R1
SC_UC723              --------ETNAYNVTTHADGTATYGPRVTK          R2
SC_AIS2002058         --------KTNAYNVTTHADGTATYGPRVTK          R3
SC_JCSC 6688          --------ETNAYNVTTHADGTATYGPRVTK          R3
SC_AIS2002055         --------KTNAYNVTTHADGTATYGPRVTK          R4
SC_UC18               --------ETNAYNVTTHADGTATYGPRVTK          R4
SC_AIS2002054         --------KTNAYNVTTHADGTATYGPRVTK          R4
SC_UB637              --------ETNAYNVTTHADGTATYGPRVTK          R4
SC_LQ41               --------KTNAYNVTTHADGTATYGPRVTK          R4
SC_UC304              --------ETNAYNVTTHADGTATYGPRVTK          R4
SC_NCTC 8325          --------KTNAYNVTTHADGTATYGPRVTK          R5
SC_IFH556             --------KTNAYNVTTHADGTATYGPRVTK          R5
SC_MW2                --------KTNAYNVTTHADGTATYGPRVTK          R5
SC_MMR-V              --------ETNAYNVTTHADGTATYGPRVTK          R5
SC_19                 --------ETNAYNVTTHADGTATYGPRVTK          R5
SC_UCL341             --------ETNAYNVTTHADGTATYGPRVTK          R5
SC_SH751              --------ETNAYNVTTHADGTATYGPRVTK          R5
SC_JCSC 7623          --------ETNAYNVTTHADGTATYGPRVTK          R5
SC_Dresden-275757     --------ETNAYNVTTHADGTATYGPRVTK          R5
SC_S_2023.LVRQ.AN     --------KTNAYNVTTHADGTATYGPRVTK          R5
SC_UE1109             --------ETNAYNVTTHADGTATYGPRVTK          R5
SC_UE1097             --------KTNAYNVTTHADGTATYGPRVTK          R5
SC_213                --------ETNAYNVTTHADGTATYGPRVTK          R6
SC_SG03               --------KTNAYNVTTHADGTATYGPRVTK          R6
SC_Stp-12             --------ETNAYNVTTHADGTATYGPRVTK          R6
SC_SH60               --------ETNAYNVTTHADGTATYGPRVTK          R6
SC_JCSC6074           --------ETNAYNVTTHADGTATYGPRVTK          R6
SC_JCSC1469           --------KTNAYNVTTHADGTATYGPRVTK          R6
SC_JCSC6075           --------ETNAYNVTTHADGTATYGPRVTK          R6
SC_JCSC6669           --------ETNAYNVTTHADGTATYGPRVTK          R6
SC_AH1                --------KTNAYNVTTHADGTATYGPRVTK          R6
SC_67-013             --------KTNAYNVTTHADGTATYGPRVTK          R6
SC_SH640              --------ETNAYNVTTHADGTATYGPRVTK          R6
SC_01093              --------ETNAYNVTTHADGTATYGPRVTK          R6
SC_IRQBAScoa28        --------KTNAYNVTTHADGTATYGPRVTK          R6
SC_JCSC6665           --------KTNAYNVTTHADGTATYGPRVTK          R6
SC_UE1112             --------ETNAYNVTTHADGTATYGPRVTK          R6
SC_17573              --------KTNAYNVTTHADGTATYGPRVTK          R6
SC_85/2232            --------KTNAYNVTTHADGTATYGPRVTK          R6
SC_SH488              --------ETNAYNVTTHADGTATYGPRVTK          R6
SC_Tager 104          --------ETNAYNVTTHADGTATYGSRVTK          R7
SC_NCTC13712          --------ETNAYNVTTHADGTATYGPRVTK          R7
SC_104                --------ETNAYNVTTHADGTATYGPRVTK          R7
SC_S_4031.LGMP.AI     --------KTNAYNVTTHADGTATYGPRVTK          R7
SC_SH407              --------KTNAYNVTTHADGTATYGPRVTK          R7
SC_4074               --------KTNAYNVTTHADGTATYGPRVTK          R7
SC_M9N                --------KTNAYNVTTHADGTATYGPRVTK          R7
SC_JCSC6908           --------ETNAYNVTTHADGTATYGPRVTK          R7
SC_BB                 --------KTNAYNVTTHADGTATYGPRVTK          R8
SC_JCSC 7552          --------KTNAYNVTTHADGTATYGPRVTK          R8
SC_JCSC 7619          --------KTNAYNVTTHADGTATYGPRVTK          R8
SC_IFH467             --------ETNAYNVTTHADGTATYGPRVTK          R8
SC_STP25              --------KTNAYNVTTHADGTATYGPRVTK          R8
SC_NCTC7972           --------KTNAYNVTTHADGTATYGPRVTK          R8
SC_JCSC 7553          RPTYKKPSETNAYNVTTHADGTATYGPRVTK          R9
SC_Ku                 RPTQNKPSKTNAYNVTTHADGTATYGPRVTK          R9
                      ***  *** **********************
                      ------→←--- R9 / C-t cap ---→
                      RPTQNKPSKTNAYNVTTHADGTATYGPRVTK
                         YK    E
```

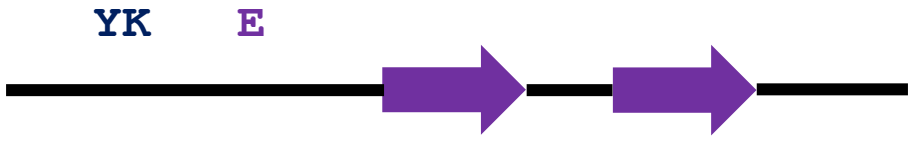

Supplemental Figure S1. Structure-based alignment of staphylocoagulase (SC) C-terminal repeat domains.

Sequences included in the alignment highlight the variation observed in the bacterial ProT activator. The sequences are clustered according to the number of repeats within the RD2 domain, as indicated to the right. Individual repeats are numbered sequentially (R1–R9). The boundaries of the repeats follow the register definition introduced by Dr. Höök and colleagues (Ko et al. 2016), which corresponds to the predicted secondary structure (β-strands, represented as arrows). Note that all RD2 repeats comprise 27 residues, except for the almost strictly conserved C-terminal cap, which is four-residue shorter. Strictly conserved residues are indicated with asterisks. The consensus sequence for each repeat is provided below the alignment; residues that deviate from this "canonical" sequence are indicated. Note that sequence variation is primarily clustered within the β-hairpin and the C-terminal end of the inter-repeats loop. "L" denotes the linker connecting the RD1 and RD2 modules.

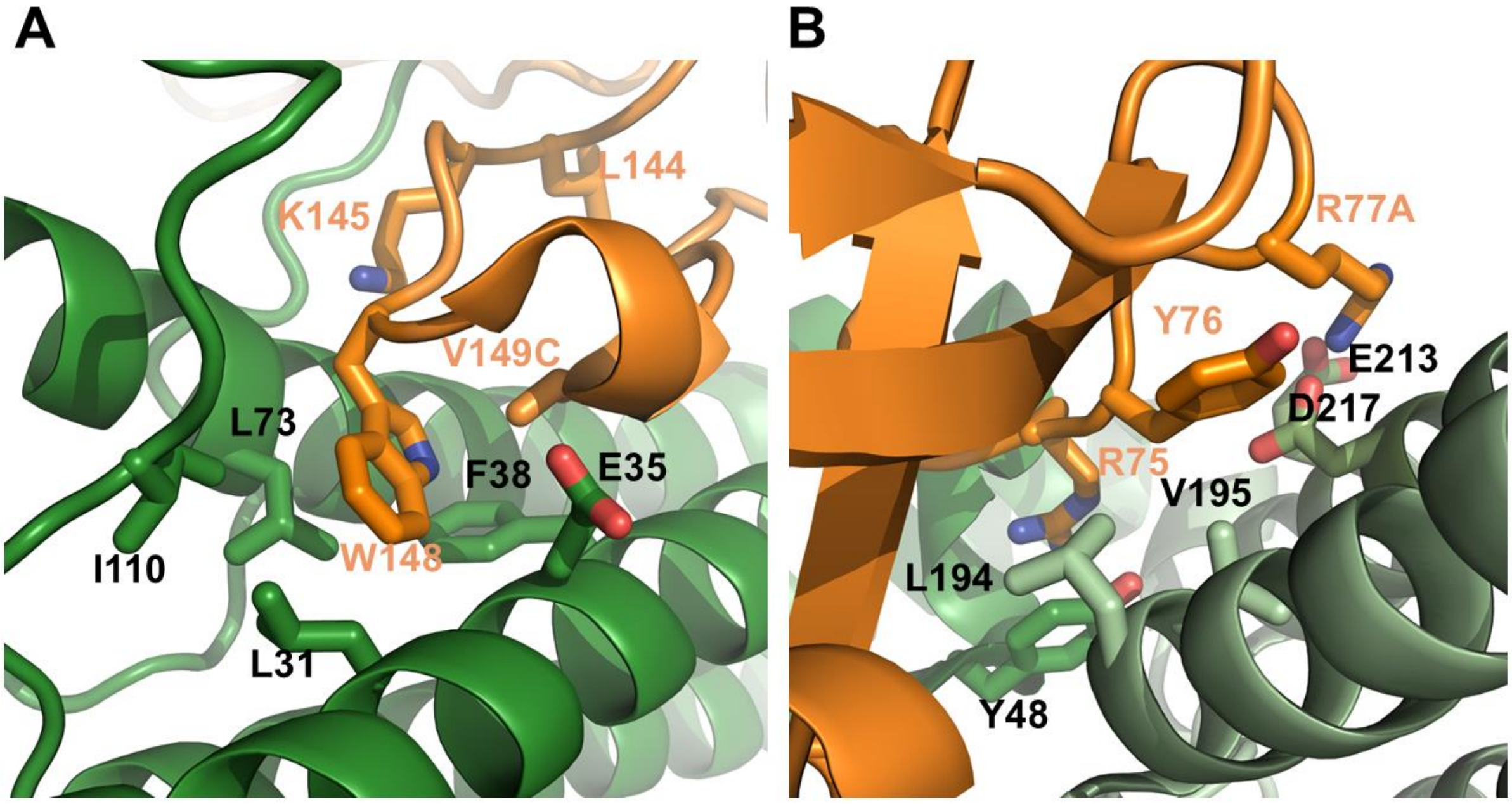

Supplemental Figure S2. Molecular details of SC N-terminal domains in complex with prethrombin-2.

(A) Interaction between domain D1 and the 148-loop (autolysis loop). The prominent Trp^{148} side chain is sequestered within a hydrophobic cradle formed by aliphatic residues from D1 helices α1 and α2, supplemented by thrombin's Val^{149C}. Consequently, Trp^{148} is displaced by ~10 Å from its position in free, active-site inhibited α-thrombin. The side chains of the "gatekeeper" residues, Leu^{144} and Lys^{145} are also displayed. Replacement of these two residues by bulkier, basic arginines might explain the SC's failure to activated bovine ProT.

(B) Detailed view of exosite I interactions. For clarity, only the side chains of three thrombin-specific residues within the 70-80 loop (Arg^{75}, Tyr^{76} and Arg^{77A}) are shown alongside the complementary interacting residues provided mainly by domain D2. Similar interactions are expected in the SC·ProT* complex, as exosite I is fully solvent exposed in the full-length zymogen.

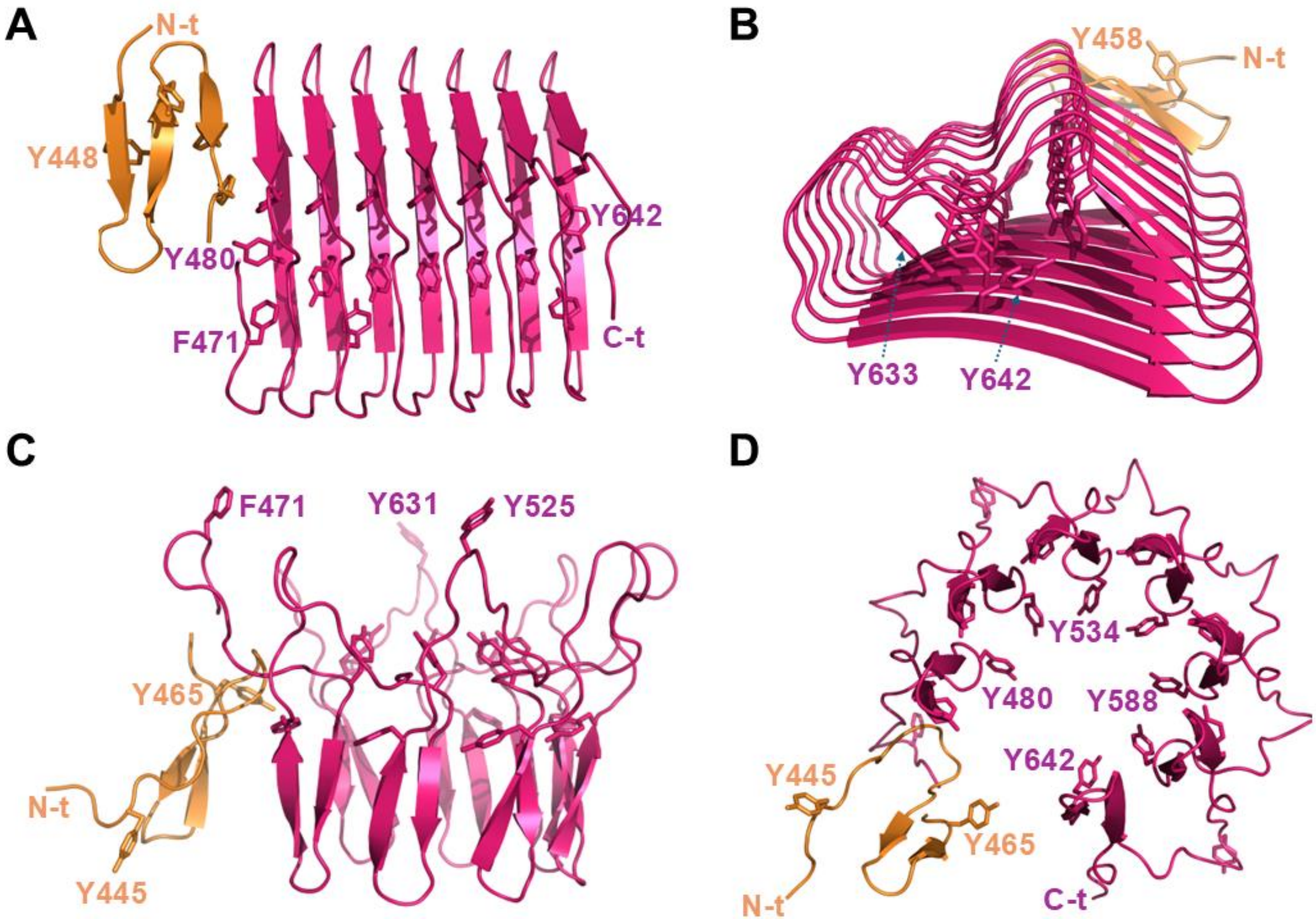

Supplemental Figure S3. Alternative structural models of SC-RD2.

In addition to the single-chain β-sheet (SLB), state-of-the-art structure prediction algorithms generate two distinct alternative β-strand arrangements for SC-RD2: a β-sandwich and an open barrel. Aromatic side chains are displayed to facilitate comparison of the hydrophobic cores and inter-repeat interfaces.

(A, B) The β-sandwich arrangement predicted by Boltz-2 shown in two approximately perpendicular views. Note the two "ladders" of Tyr residues packed within the protein core. Such an arrangement is generally considered energetically unfavorable; structurally characterized β-sandwich domains typically feature buried aliphatic residues (Ile, Val, Leu) to stabilize the hydrophobic core.

(C, D) Open barrel conformation predicted by RoseTTAFold. In this model, the β-hairpins remain essentially independent. The barrel-like assembly is stabilized primarily by hydrogen bonds between the hydroxyl group of a Tyr residue in one repeat and a carbonyl oxygen atom from the succeeding repeat.

A

```
           480    .  490    .  500    .  510    .  520    .  530    .  540    .  550    .
SC         ETNAYNVTTHANGQVSYGARPTQNKPSKTNAYNVTTHGNGQVSYGARPTYKKPSKTNAYNVTTHA-NGQVSYGARPTQNKPS
         950    .  960    .  970          .  980   .  990           . 1000    .
CPAP       LPDGGRLVVFPNGTRKELSADG--------QTVKVMFFNGDVKHTMPDQ---------RVIYYYAEAQTTHITYPDG-----
           ←---------- R1 ---------→←---------- R2 ---------→←----------- R3 -----------→

          560    .  570    .  580    .  590    .  600    .  610    .  620    .  630    .
SC         KTNAYNVTTHGNGQVSYGARPTQNKPSKTNAYNVTTHGNGQVSYGARPTQNKPSKTNAYNVTTHANGQVSYGARPTYKKPS
          1010    . 1020    .          1030    . 1040    .          1050    . 1060
CPAP       ----MEVLQFPNNQTEKHFPDG---------RKEITFPDQTVKTLHPDG---------REESVLTDGTIIQLNPDG-----
           ←---------- R4 ---------→←---------- R5 ---------→←---------- R6 ---------→

           640    .  650    .  660
SC         ETNAYNVTTHADGTATYGSRVTK
               . 1070    . 1080
CPAP       ----SKVIQFNTGQREIHTADFK
           ←-------- R7 --------→
```

B

```
           480    .  490    .  500    .  510    .  520    .  530    .  540    .  550    .
SC         ETNAYNVTTHANGQVSYGARPTQNKPSKTNAYNVTTHGNGQVSYGARPTYKKPSKTNAYNVTTHANGQVSYGARPTQNKPS
           160    .  170    .      180    .  190    .  200        .  210    .  220       .
MORN1      KMHGHGKYIYSDGGVYEGDW----IDGKMHGKGTYVFPNGNVYEGEW----AHDMKDGYGVLTYQNGEKYEGYW----KQD
           ←---------- R1 ---------→←---------- R2 ---------→←---------- R3 ----------→

          560    .  570    .  580    .  590    .  600    .  610    .  620    .  630    .
SC         KTNAYNVTTHGNGQVSYGARPTQNKPSKTNAYNVTTHGNGQVSYGARPTQNKPSKTNAYNVTTHANGQVSYGARPTYKKPS
          230    .  240    .      250    .  260    .  270        .  280    .  290      .
MORN1      KVHGKGTLTYTRGDKYIGDW----MDAKKDGEGELIYANGDRFKGQWADD----RANGFGVFTYANGNRYEGEW-TDDKR-
           ←---------- R4 ---------→←---------- R5 ---------→←---------- R6 ---------→

           640    .  650    .  660
SC         ETNAYNVTTHAD-GTATYGSRVTK
             300    .  310    .
MORN1      --HGRGVFYCAEDGSAYEGEFVGG
           ←-------- R7 --------→
```

Supplemental Figure S4. Structure-based sequence alignment of SC RD2 and topologically equivalent domains in MORN superfamily proteins. (**A**) TCP10 domain of *Danio rerio* CPAP (MCPH6). (PDB: 4BXR; aligned from the complex with the STIL/MCPH7 peptide). (**B**) *Toxoplasma gondii* MORN1 protein (PDB: 6T69). Strictly conserved residues are shown in white text with green shading; conservative replacements are shaded gray. Note the low sequence similarity, particularly with the vertebrate homolog, which contrasts with the significant degree of structural similarity observed in Figure 4.

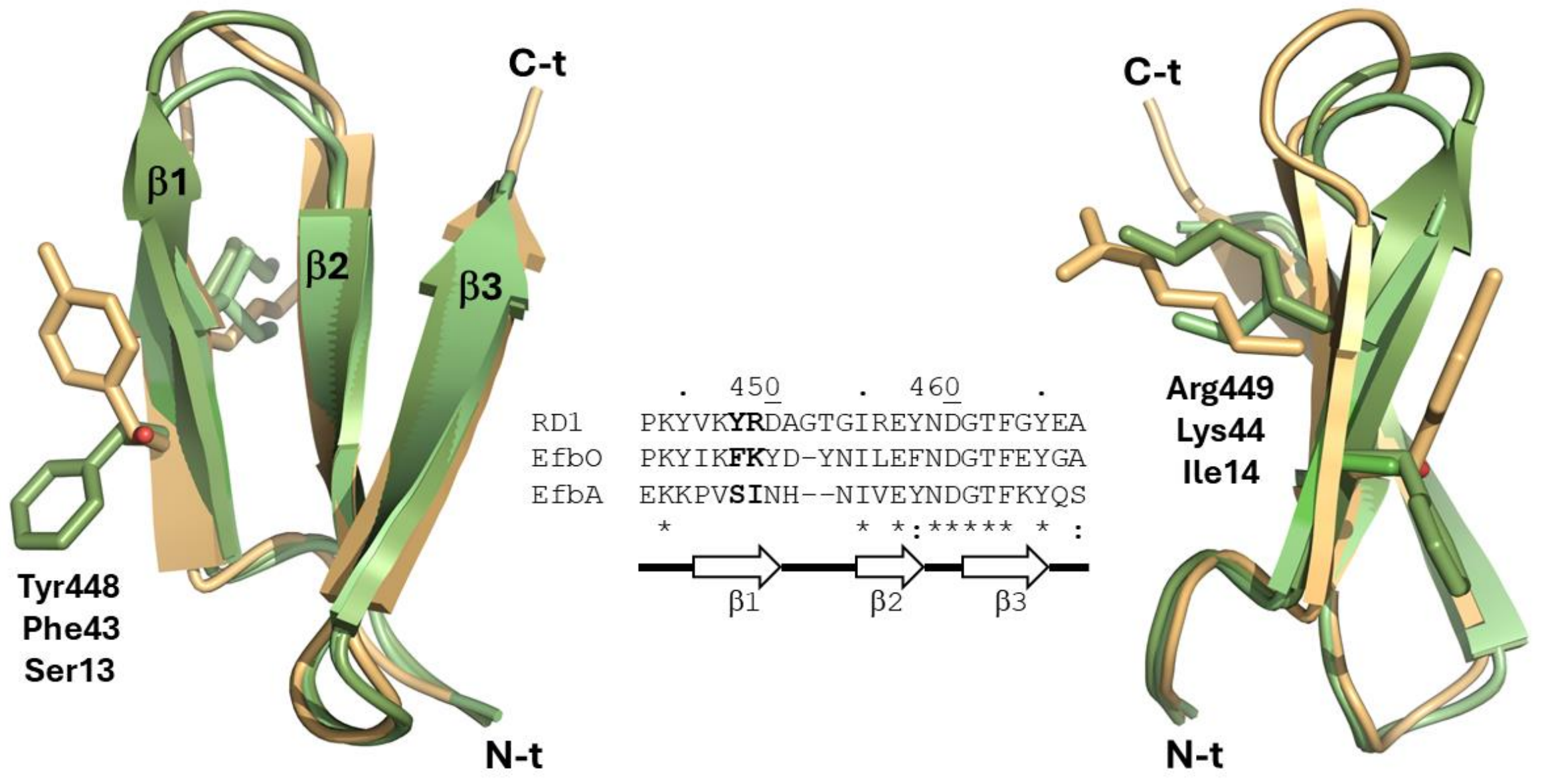

Supplemental Figure S5. **Structural comparison of SC-RD1 and homologous Efb modules.**

3D models of the SC-RD1 domain and the sequence-related modules of Efb (Efb-A and Efb-O) are shown as superimposed cartoons. All three domains share a conserved three-stranded β-sheet framework, and nearly identical C-terminal sequences. However, they exhibit striking differences in their N-terminal regions, particularly in the length and sequence of the β1-β2 hairpin. While SC and Efb share a common binding site on Fbg (Palma et al. 2001; Ko et al. 2016; Bertoglio et al. 2023), Efb-O displays a 200-fold higher affinity for Fbg than Efb-A (Ko et al. 2011). A comparison of the modules reveals a critical substitution at the predicted interface with hole 'b': the major aromatic residue Tyr^{448} (SC numbering), is conservatively replaced by Phe in high-affinity Efb-O, whereas the topologically equivalent residue in lower-affinity Efb-A is a small, polar serine. This, alongside other non-conservative exchanges in strand β1, suggests that these residues form an essential Fbg-binding epitope and lend strong support to our current RD1-Frag D model (compare with Figure 5).

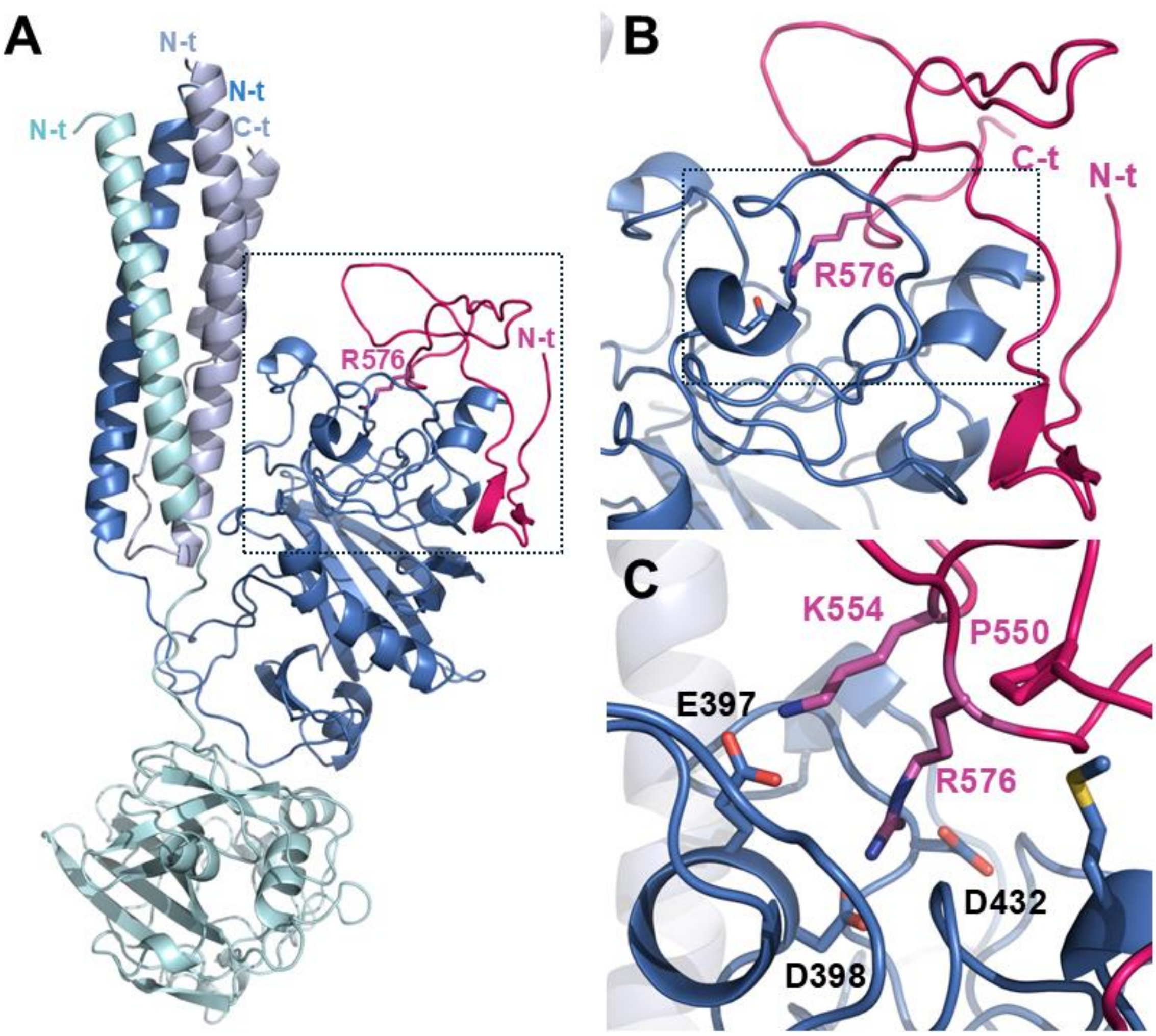

Supplemental Figure S6. **The basic inter-repeat loops in SC-RD2 might engage holes 'a' or 'b' in fibrinogen fragment D.**

(**A**) 3D model of a putative complex between SC repeat R4 and Frag D. Note that these Arg-mediated interactions are not compatible with the folded conformations of SC-RD2, either in the SLB architecture (Figure 3) or in the β-sandwich or barrel arrangements (Supplemental Figure S3).

(**B**) Close-up showing the SC repeat inserted into the hole 'b' of Frag D. In this extended conformation, other repeats can engage Frag D moieties form the same or another fibrinogen molecule, resulting in the observed stoichiometries of 3-5 mol of Fbg per mol SC.

(**C**) Major interactions at the interface between the SC repeat and hole 'b'. The Arg^{576} side chain extends into the highly acidic pocket of the b chain and forms strong H-bonded slat bridges with the Asp^{398} carboxylate.

Supplementary Table 1: Structurally characterized homologs of SC single-layer β-sheet (SLB).

Protein / Organism	Function	PDB	Seq. Id.	E-value
MORN1 / *Trypanosoma brucei*	Involved in endocytosis. Regulates flow of macromolecular cargo through the neck of the flagellar pocket.	6T4R, 6T68	16.6%	3.1e-6
MORN1 / *Toxoplasma gondii*	Critical scaffolding role, specifically organizing the basal complex and facilitating daughter cell formation. Essential for *T. gondii* cell division.	6T6Q, 6T69	20.2%	1.8e-5
MORN1 / *Plasmodium falciparum*	Involved in endocytosis and in regulating the flow of macromolecular cargo through the neck of the flagellar pocket.	6T4D	16.2%	2.0e-5
Centromere protein J (CenpJ/CPAP) / *Danio rerio*	Plays a role in centriole elongation. Binds highly conserved Pro-rich motif in STIL.	4BXR, 4LD3[1]	13.0%	1.9e-3
Spindle assembly abnormal 4 (Sas-4/ dCPAP) / *Drosophila melanogaster*	Centriole/centrosome assembly. Binds Pro-rich motif in anastral spindle 2 (Ana2/STIL).	4BY2, 4MPZ	12.7%	1.3e-3
Radial spoke protein 10 (FAP266) / *Chlamydomonas reinhardtii*	Structural component of the axonemal radial spokes in the flagella (cilia).	7SQC	16.1%	4.1e-3
Radial spoke protein 1 (RSP1) / *C. reinhardtii*	Part of the radial spoke complex. Binds the C-terminal peptide of RSP4.	7JR9[2]	13.9%	6.6e-3
Central apparatus associated protein C1a-18 (FAP277) / *C. reinhardtii*	Part of the ciliary C1 central pair apparatus. Binds a central stretch of protein C1a-34.	7N6G, 7SQC	13.6%	2.7e-1
Radial spoke head 1 homolog / *Mus musculus*	Part of radial spoke complexes. Plays an important role in the motility of sperm	7DMP, 8WZB, 8X2U	14.2%	1.5e-2

[1] In addition to entries 4BXP, 4BXQ, 4LD1, and 4LZF.
[2] In addition to entries 7JRJ, 7JTK, and 8GLV.

	and cilia. Binds the C-terminal peptide of Rsph4a.			
Radial spoke head 1 homolog (RSPH1) / *Homo sapiens*	Part of the 96-nm repeat of human respiratory doublet microtubule and associated axonemal complexes that play an important part in the motility of sperm and cilia.	8J07	12.0%	1.3e-2
MORN4 / *Mus musculus*	Plays a role in promoting axonal degeneration following neuronal injury by toxic insult or trauma. Binds with high affinity to the Myo3a tail.	6JLE	15.6%	1.4e-1
Outer surface protein (OspA) / *Borreliella (Borrelia) burgdorferi*	Major immunogenic protein of the Lyme disease spirochete. Facilitates spirochete colonization of the vector's gut by binding to the tick receptor of OspA (TROSPA). Binds CD40 on brain microvascular endothelial cells allowing neuroinvasive *Borrelia* strains to cross the brain-blood barrier.	3CKA, 3EEX[3]	13.9%	2.6e-1

Data were obtained via FoldSeek; for entries where multiple structural alignments were possible, the highest values of sequence identity and lowest E-value are reported. Several of these homologs are known to form homodimers or higher-order multimers, most notably those from protozoans parasites.

[3] In addition to over 50 reported crystal structures of natural or engineered OspA variants, either free or complexed with specific antibodies (1FJ1, 1OSP, 2AF5, 2FKG, 2FKJ, 2G8C, 2HKD, 2I5V, 2I5Z, 2OL6, 2OL7, 2OL8, 2OY1, 2OY5, 2OY7, 2OY8, 2OYB, 2PI3, 3AUM, 3CKF, 3CKG, 3EC5, 5B2A, 5YS7, 5Z1O, 6AIS, 6ICS, 6IDC, 6IEI, 6IYS, 6J47, 6J48, 6J49, 6J5M, 6J5N, 6J5O, 6J5P, 6J5Q, 6J5R, 6J6B, 6J6C, 6J6D, 6J6E, 6KK9, 6KT1, 6KWJ, 6KWU, 6KWV, 6LJY, 7FDD, 7FJR, 7JWG, 7T25, 8TUZ, 8TV3, 8TVD, 8TVJ, 8XWG, 9BYF, 9CDS, and 9JSA).
